# Mucosal-Associated Invariant T Cells Develop an Innate-Like Transcriptomic Program in Anti-mycobacterial Responses

**DOI:** 10.3389/fimmu.2020.01136

**Published:** 2020-06-09

**Authors:** Manju Sharma, Shuangmin Zhang, Liang Niu, David M. Lewinsohn, Xiang Zhang, Shouxiong Huang

**Affiliations:** ^1^Division of Environmental Genetics and Molecular Toxicology, Department of Environmental and Public Health Sciences, University of Cincinnati College of Medicine, Cincinnati, OH, United States; ^2^Division of Biostatistics and Bioinformatics, Department of Environmental and Public Health Sciences, University of Cincinnati College of Medicine, Cincinnati, OH, United States; ^3^Pulmonary & Critical Care Medicine, Oregon Health & Science University, Portland, OR, United States; ^4^Genomics, Epigenomics and Sequencing Core, Department of Environmental and Public Health Sciences, University of Cincinnati College of Medicine, Cincinnati, OH, United States; ^5^Immunobiology Graduate Program, Cincinnati Children's Hospital, Cincinnati, OH, United States

**Keywords:** mucosal-associated invariant T (MAIT) cells, transcriptome, MHC-related protein 1 (MR1), *Mycobacterium tuberculosis*, innate-like activation

## Abstract

Conventional T cells exhibit a delayed response to the initial priming of peptide antigens presented by major histocompatibility complex (MHC) proteins. Unlike conventional T cells, mucosal-associated invariant T (MAIT) cells quickly respond to non-peptidic metabolite antigens presented by MHC-related protein 1 (MR1). To elucidate the MR1-dependent activation program of MAIT cells in response to mycobacterial infections, we determined the surface markers, transcriptomic profiles, and effector responses of activated human MAIT cells. Results revealed that mycobacterial-incubated antigen-presenting cells stimulated abundant human CD8^+^ MAIT cells to upregulate the co-expression of CD69 and CD26, as a combinatorial activation marker. Further transcriptomic analyses demonstrated that CD69^+^CD26^++^ CD8^+^MAIT cells highly expressed numerous genes for mediating anti-mycobacterial immune responses, including pro-inflammatory cytokines, cytolytic molecules, NK cell receptors, and transcription factors, in contrast to inactivated counterparts CD69^+/−^CD26^+/−^ CD8^+^MAIT cells. Gene co-expression, enrichment, and pathway analyses yielded high statistical significance to strongly support that activated CD8^+^ MAIT cells shared gene expression and numerous pathways with NK and CD8^+^ T cells in activation, cytokine production, cytokine signaling, and effector functions. Flow cytometry detected that activated CD8^+^MAIT cells produced TNFα, IFNγ, and granulysin to inhibit mycobacterial growth and fight mycobacterial infection. Together, results strongly support that the combinatorial activation marker CD69^+^CD26^++^ labels the activated CD8^+^MAIT cells that develop an innate-like activation program in anti-mycobacterial immune responses. We speculate that the rapid production of anti-mycobacterial effector molecules facilitates MAIT cells to fight early mycobacterial infection in humans.

## Introduction

Innate and adaptive immune systems have framed a classical dichotomy of immune responses in health and diseases. Innate immune cells rapidly recognize and respond to microbial and endogenous ligands with germline-encoded innate receptors (1). In contrast, conventional naïve T cells undergo a prolonged clonal expansion in responses to major histocompatibility complex (MHC)-presented peptide antigens (1, 2). It has been demonstrated that CD4^+^ and CD8^+^ T cells are crucial in combating mycobacterial infections in T cell-deficiency and adoptive transfer mouse models (3, 4). In humans, CD4^+^ T cells are essential in fighting mycobacterial infections evidenced by a much higher risk of active tuberculosis in HIV coinfection with defective CD4^+^ T cell responses (5) and conventional CD8^+^ T cells suppress mycobacterial growth (4). However, a large number of mycobacterial-reactive CD8^+^ T cells in humans are activated by non-peptidic antigens presented by MHC class I-related protein I (MR1) and are identified as mucosal-associated invariant T (MAIT) cells (6–12). Therefore, labeling the mycobacterial-reactive MAIT cells and defining their activation program regarding its similarities to conventional T cell or innate cells will facilitate the understanding of their unique activation pathways and effector functions in anti-mycobacterial responses.

Previous observations support that MAIT cell response is “innate-like” upon the stimulation with mouse (8–12) and bacterial antigens (6–8), as different from conventional T cells. The fundamental explanation of this “innate-like” MAIT cell activation initially bases on the evidence that MAIT cells express an invariant TCRα chain to interact with a conserved metabolite antigen presented by a monomorphic MR1 protein (12–17). This conservation interestingly contrasts to the tri-molecular interaction for conventional T cell activation using heterogeneous TCRs to recognize variable peptide antigens presented by heterogeneous HLA molecules (1, 2). In a new bacterial infection, MAIT cells can be instantly activated by MR1-mediated antigen presentation bypassing a long priming stage for conventional T cells (8–12). Recently, MAIT cells were demonstrated to protect mice against mycobacterial infections (8, 18) and respond to mycobacterial-infected human cells (7, 8). Surprisingly, abundant MAIT cells in uninfected and latently infected individuals exhibit strong reactivity to mycobacteria and potentially complement conventional T cells in anti-mycobacterial defenses (7, 8). Moreover, reduced frequency of MAIT cells in active tuberculosis (7, 8, 19, 20) seriously demands the elucidation of MAIT cell activation programs and effector responses in mycobacterial infections. Although riboflavin precursor metabolites have been identified from multiple non-tuberculous bacteria (13, 16, 17) and the general activation program of MAIT cells has been defined using *E.coli*, cytokine, or anti-CD3 stimulation (21–26), it remains uncertain whether mycobacteria efficiently activate human MAIT cells and which surface markers specifically label activated MAIT cells in mycobacterial infection. Moreover, whether activated human MAIT cells show an innate-like transcriptomic program to elicit strong anti-mycobacterial immune responses is unknown. Therefore, applying MAIT cell-specific stimulants, defining the activation program, and determining the effector responses of human MAIT cells in mycobacterial infections will be highly translational to clinically label MAIT cells and mechanistically understand the anti-mycobacterial immune responses of MAIT cells in diseases.

In this study, we elucidated the activation programs of MAIT cells in mycobacterial infections to determine their similarity to CD8^+^ T and natural killer (NK) cells. Specifically, we activated the dominant CD8^+^MAIT cell population with mycobacterial stimulation and used the combinatory marker CD69^+^CD26^++^ to separate activated MAIT cells for transcriptomic analyses. Results revealed that CD69^+^CD26^++^ CD8^+^MAIT cells shared key gene expression and activation pathways with conventional CD8^+^ T cells and NK cells, supporting a unique “innate-like” activation program to induce early anti-mycobacterial responses.

## Materials and Methods

### Preparation of Human Monocyte-Derived Dendritic Cells and MR1-Overexpressing Cells

Blood samples free of detectable infectious or non-infectious diseases were obtained from healthy donors with written informed consent at the Hoxworth Blood Center in the University of Cincinnati. De-identified blood samples were processed according to the protocols approved by the Institutional Review Board of the University of Cincinnati. We isolated human peripheral blood mononuclear cells (PBMCs) and prepared human monocyte-derived dendritic cells (MoDCs) as performed (27, 28). Briefly, upon the isolation of PBMCs using Ficoll-paque gradient (GE Healthcare), we enriched monocytes by plastic adherence and differentiated monocytes using human recombinant granulocyte-macrophage colony-stimulating factor (300 U/ml) and interleukin 4 (200 U/ml) for 5 days (27, 28). The matured MoDCs were further activated with lipopolysaccharide (LPS) at 50 ng/ml for 24 h or incubated with bacteria. We also overexpressed the human MR1 protein by transducing the wild type human MR1 gene with the retroviral vector pMXIP (12) into human HLA-defective myeloid cell line K562 (K562.hMR1) (29) as we reported (11). The expression of the recombinant human MR1 protein was confirmed with flow cytometry, and its function was tested by MAIT cell line activation in bacterial incubation.

### Bacterial Incubation of Antigen-Presenting Cells

MoDCs and K562.hMR1 cells were incubated with *Listeria monocytogenes (L. monocytogenes* strain J0161, Bei resources*), Escherichia coli (E. coli* strain BL21, New England BioLabs), *Mycobacterium bovis-* (*M. bovis-*) derived Bacille Calmette-Guerin (BCG) vaccine (Pasteur strain) (30), and avirulent *Mycobacterium tuberculosis* (*M. tuberculosis*, strain H37Ra, Colorado State University, Fort Collins, CO) (31). Both mycobacterial strains BCG and H37Ra were cultured for 5–6 days in middlebrook 7H9 complete medium at 37°C using an orbital shaker with a speed setting at 270 rpm. *E.coli* and *L. monocytogenes* were cultured overnight at 37°C in the Luria-Bertani broth using an orbital shaker at 100 rpm. Bacteria were harvested at a log-growing phase, washed with phosphate buffer saline (PBS), and measured for their absorbance (optical density at wavelength 600 nanometres, OD_600_) according to the report (32). OD_600_ provides a semi-quantitative method to estimate bacterial cell numbers sufficient for MAIT cell activation (32). Human MoDCs or K562.hMR1 cells were incubated with *E.coli* in an estimated cell to bacteria ratio of 1:5 and 1:40 and with BCG in a ratio of 1:0 and 1:100. The blockage of activation was performed with an anti-MR1 antibody (clone 26.5, mouse IgG2a, at 2 μg/ml) that blocks MR1-dependent MAIT cell activation (10–12). Anti-HLAI antibody (clone W6/32, mouse IgG2a, Biolegend, at 2 μg/ml) was used as an isotype control for the anti-MR1 antibody and was also used to block the irrelevant effect of MHC class I proteins with similar structures as MR1 (33). Moreover, the chemical inhibitor cyclosporine A (CsA), mainly blocking TCR-mediated calcium signaling pathway for T cell activation (34, 35), was applied at 0.5 μg/ml.

### Enzyme-Linked Immunospot

Upon incubation with bacteria overnight, MoDCs and K562.hMR1 cells were washed and incubated with the MAIT cell line (D466F5) (7) in a ratio of 5:1 and 1:4, respectively, by considering the estimated sizes of these cell types for optimal cell contact. The enzyme-linked immunospot (ELISPOT) assay was performed, as we reported (27). Briefly, both bacterial-incubated antigen-presenting cells and MAIT cells were co-cultured for 5 or 15 h on the multiscreen filter plate (Millipore) coated with anti-human IFNγ antibody (Mabtech). IFNγ^+^ MAIT cell spots were then developed with an indirect immunostain approach using a biotinylated anti-human IFNγ antibody (Mabtech), ExtraAvidin conjugated by alkaline phosphatase (Sigma), and substrates BCIP/NBT (Sigma). We used CTL-ImmunoSpot S6 Micro Analyzer to visualize and quantify IFNγ^+^ MAIT cell spots. Directional differences between bacterial-incubated and non-incubated conditions and between without and with anti-MR1 blockage were statistically analyzed using a paired *t*-test.

### Isolation and Activation of Primary Human MAIT Cells

Upon removing adhered monocytes, human PBMCs were incubated with anti-Vα7.2 antibody (3C10) conjugated with PE (Biolegend) and followed by a positive selection with anti-PE antibody-conjugated magnetic beads (MACS, Miltenyi Biotec), according to the manufacturer's instructions. Bacterial-incubated K562.hMR1 cells were washed and further incubated with anti-Vα7.2-enriched PBMCs with abundant primary human MAIT cells in a ratio of 1:4 for 15 h with the presence of an anti-CD28 (clone CD28.2) antibody at 2 μg/ml. In addition to the stimulation with bacterial-incubated MR1-expressing K562 cells, anti-Vα7.2-enriched (MAIT cell-enriched) and anti-Vα7.2-depleted (conventional T cell-enriched) PBMCs were also activated with pre-coated anti-CD3 (clone UCHT1) at 1 μg/ml and anti-CD28 (clone CD28.2) at 2 μg/ml as positive controls. Hence, anti-CD28 (clone CD28.2) at 2 μg/ml was used for all K562.hMR1- and anti-CD3-mediated stimulation of T cells. For intracellular cytokine staining, Brefeldin A (10 μg/ml), which inhibits protein transport from the endoplasmic reticulum to the Golgi complexes, was added 2 h prior to cell harvesting for intracellular staining.

### Antibodies and Flow Cytometry for Human MAIT Cells

Upon the co-culture for 15 h, surface markers, intracellular cytokines, and transcription factors of MAIT cells were stained following the manufacturer's instructions (Biolegend unless noted). In brief, cells were washed twice with staining buffer (PBS with 2% FBS) then blocked with anti-human Fc receptor antibodies, including anti-CD64 (mouse IgG1, clone 10.1), CD32 (mouse IgG2b, clone FUN-2), CD16 (mouse IgG1, clone 3G8), and additional Fc receptor blocking solution human TruStain FcX. For surface staining, cells were incubated with the combination of monoclonal antibodies, including phycoerythrin (PE)-Vα7.2, biotin-CD4 (OKT4) and streptavidin-conjugated quantum dot 525 or brilliant violet 510-CD4, brilliant violet 711-CD8α (RPA-T8), Allophycocyanin/Cyanine7 (APC/Cy7)-CD161 (HP-3G10), brilliant violet 605 or 421-CD69 (FN50), and PE/Cy5-CD26 (BA5b), for 30 min at 4°C in dark. PE-CD3 (OKT3) was used to replace PE-Vα7.2 for Vα7.2-depleted T cells. The MR1 tetramer loaded with the *E.coli* metabolite 5-amino-6-D-ribitylaminouracil (5-A-RU) (16, 36) and labeled with brilliant violet 421 was obtained from the NIH tetramer facility. For the staining of intracellular cytokines and transcription factors, cells were first incubated with antibodies against surface markers. Then, cells were fixed and permeabilized using the Fix/Perm Kit (Biolegend) and further stained in the 1 x Perm buffer for 30 min on ice with anti-cytokine and anti-transcription factor antibodies, including PE/Cy7-TNF-α (MAb11), APC-IFNγ (4S.B3), Alexa fluor 647-granulysin (DH2), PE/Cy7-Tbet (4B10), and Alexa fluor 488-Eomes (644730, R&D systems). Flow cytometry used BD Fortessa and Millipore Guava EasyCyte 12 channel high throughput flow cytometer according to the manufacturer's instructions. Flow cytometry data were further compensated and analyzed using Millipore Guava incyte and FlowJo software programs. Directional differences between mycobacterial stimulation and Listeria control was statistically tested using a paired *t*-test.

### RNA-Seq of Human MAIT Cells

Anti-Vα7.2-enriched cells were co-cultured with BCG-incubated K562.hMR1 cells or stimulated with anti-CD3 antibody plus an anti-CD28 antibody for inducing co-stimulation in all conditions. Upon co-culture for 15 h, cells were first gated on Vα7.2^+^CD161^+^CD4^−^CD8^+^ to enrich this major MAIT cell subset and further sorted based on CD69^+^CD26^++^ and CD69^+/−^CD26^+/−^ to represent stimulated MAIT cells and non-stimulated MAIT-enriched cells. Similar to our report (27, 28), around one thousand cells were collected and lysed in the Lysis Buffer for total RNA extraction using mirVana kit (ThermoFisher, Grand Island, NY). RNA integrity was measured by a Bioanalyzer using Agilent RNA 6000 Pico Kit (Agilent, Santa Clara, CA) and showed a high quality of samples. Next, NEBNext Poly(A) mRNA Magnetic Isolation Module (New England BioLabs, Ipswich, MA) was used for polyA RNA purification. The library for RNA-seq was prepared by using NEBNext Ultra Directional RNA Library Prep kit (New England BioLabs, Ipswich, MA). During the second cDNA synthesis, dUTP was incorporated to maintain strand specificity. The library was enriched and indexed via 15 cycles of PCR. The amplified libraries, together with the negative control, were cleaned up for Bioanalyzer QC analysis. To study differential gene expression, individually indexed and compatible libraries at 15 pM total were proportionally pooled for clustering on single-read flow cells in cBot system (Illumina, San Diego, CA). The clustered libraries were sequenced to generate 51 bp reads at ~25 million per sample. RNA-seq data can be accessed at the GEO database (accession number GSE124381).

### Bioinformatic Analysis of Differentially Expressed Genes (DEGs)

Similar to our previous report (27), transcriptomic data were analyzed to identify DEGs between CD69^+^CD26^++^ CD8^+^MAIT cells and CD69^+/−^CD26^+/−^ CD8^+^MAIT-enriched cells representing activated MAIT cells and inactivated MAIT-enriched cells. The list of DEGs of MAIT cells were analyzed for the gene co-expression, enrichment, and activation pathways in comparison to innate immune cells and conventional T cells. Sequence reads were first aligned to the genome and converted to intensity counts. Resulted intensity counts were compared between CD69^+^CD26^++^ and CD69^+/−^CD26^+/−^ cells from three donors using the edgeR program on the Bioconductor R platform to identify DEGs based on the absolute fold change (>2 folds) and *p*-values (<0.05). DEGs were shown with volcano plots, and representative altered genes were shown with a heatmap generated with edgeR and ggplot2 programs on the R platform. To predict the co-expression and shared functional clusters of DEGs with innate immune cells and conventional T cells, we used a ToppCluster software package to search DEGs against several databases, including KEGG and REACTOME, and obtained multiple clusters of genes overlapped with other activated immune cells at a statistically significant level. ToppCluster uses a hypergeometric test to obtain functional enrichment (https://toppcluster.cchmc.org/) (37). Enrichment analyses were performed using the Gene Set Enrichment Analysis (GESA) software by searching multiple available expression databases according to the instruction (38). The shared gene clusters between MAIT cells and other immune cells were further input to software Cytoscape Version 3.3.0 (www.cytoscape.org/), a broadly used open-source software platform for visualizing complex networks. We also applied Cytoscape to search various databases, including PANTHER, MSigDB, KEGG, NCI Pathway, and Reactome databases, to identify comprehensive pathways for the activation of conventional T cells and NK cells. The DEGs were annotated using the representative pathways of T cell and NK cell activation.

### Mycobacterial Growth Inhibition in Antigen-Presenting Cells

The mCherry-labeled BCG was obtained from Dr. Russell's lab (39) and used to incubate MoDCs and K562.hMR1 cells at a ratio of 50:1 for 2 h. BCG-incubated cells were washed for 4 times and further co-cultured with rested or anti-CD3/CD28-activated primary MAIT cells upon magnetic enrichment with anti-Vα7.2 antibody. After overnight co-culture, we measure the remained mCherry-labeled BCG with antigen-presenting cells (% mCherry^+^ cells).

## Results

### Rapid Activation of a Human MAIT Cell Line by Dendritic Cells and MR1-Expressing Cells

**Figure 1:**
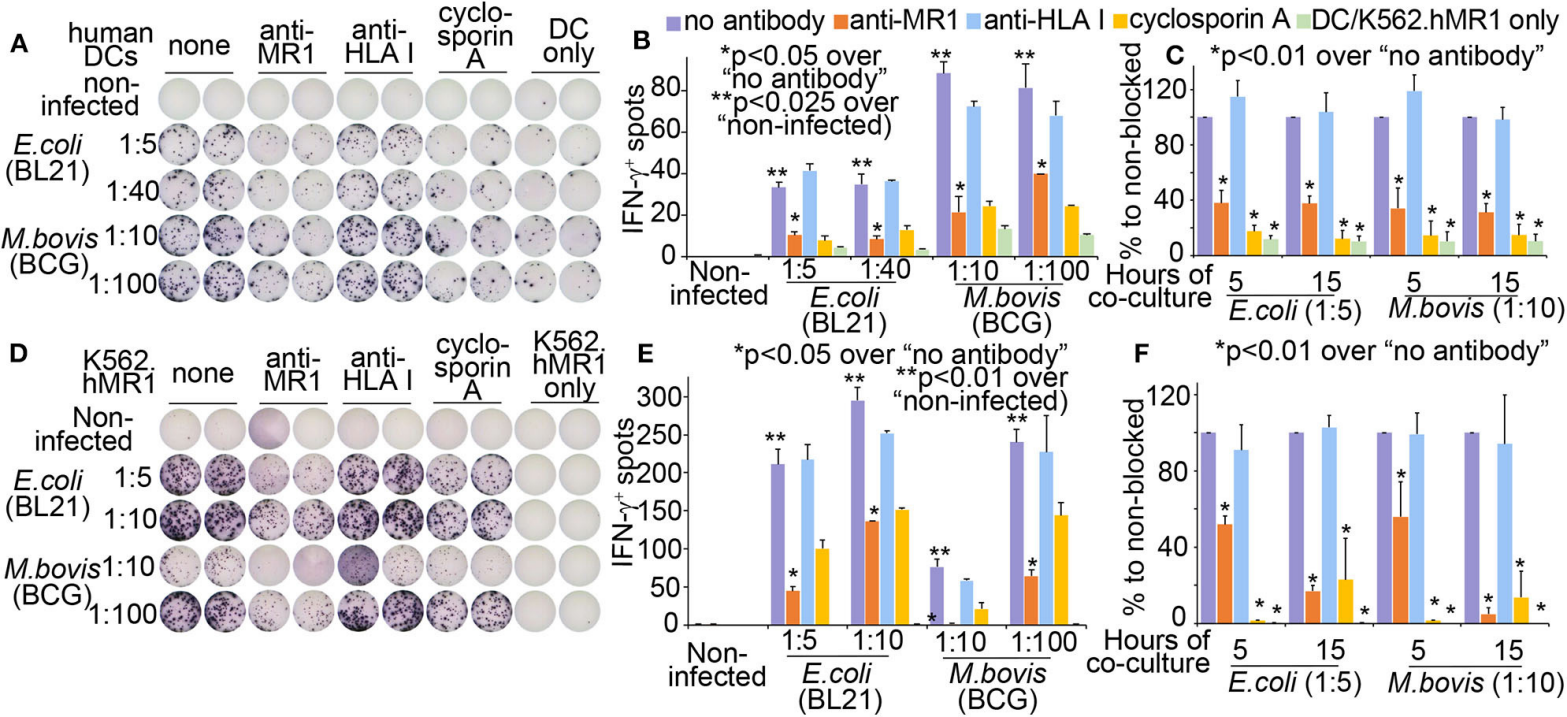
Monocyte-derived dendritic cells (MoDCs) and human MR1-overexpressed K562 cells (K562.hMR1) to activate a human MAIT cell line. Differentiated MoDCs were incubated with *E.coli* and *M. bovis* strains overnight. Bacterial cell number was estimated by OD600 absorbance and ratios of bacteria to DCs were shown. Bacterial-incubated MoDCs were washed and further co-cultured with the MAIT cell line (D466F5) (7) for 15 h. Anti-MR1 (clone 26.5, 2 μg/ml), Anti-HLAI antibody (clone W6/32 at 2 μg/ml), cyclosporine A (0.5 μg/ml), and MoDC only were used as controls. IFNγ^+^ MAIT cells measured by an ELISPOT assay were shown from one independent assay with MoDCs from one donor **(A)**. Means from technical duplicates in panel A are shown with standard errors and statistically analyzed using a paired *t*-test **(B)**. Three independent assays of DC-MAIT cell co-culture were performed with antibody blockage for 5 or 15 h. Data were normalized to non-blocked condition, shown with standard errors, and statistically analyzed using a paired *t*-test **(C)**. At the same setting to MoDCs, bacterial-incubated K562.hMR1 cells were co-cultured with the MAIT cell line (D466F5) to stimulate IFNγ^+^ spots **(D)**. IFNγ^+^ MAIT spots in technical duplicates were similarly analyzed **(E)**. Three independent assays were similarly performed and analyzed **(F)**.

Different from published monocytes (8, 24) and a lung epithelial cell line (7), antigen-presenting cells in this study used a human MR1-overexpressed human hematopoietic cell line K562 (K562.hMR1) with defective MHC expression (12, 40) and monocyte-derived dendritic cells (MoDCs) with strong co-stimulation. K562.hMR1 cells allow us to specifically detect bacterial-activated human MAIT cells and delineate MR1-dependent MAIT responses to bacterial infection, as different from bacterial-irrelevant cell activation by anti-CD3 antibody, cytokines, or autologous monocytes (22–24). MoDCs were differentiated using granulocyte-macrophage colony-stimulating factor and interleukin 4 as we reported (27, 28). Prior to the co-culture with T cells, MoDCs were overnight incubated with live *E.coli* or *M. bovis* (BCG) at indicated ratios of cell:bacteria. The bacterial-incubated MoDCs were then washed and co-cultured with a human MAIT cell line (D466F5), which was derived from MAIT cells of an active tuberculosis patient (7). As measured in an Enzyme-linked Immunospot (ELISPOT) assay, the number of IFNγ^+^MAIT cell spots significantly enhanced upon the overnight stimulation (15 h of co-culture) of BCG- and *E.coli*-incubated MoDCs, to an extent much higher than the non-infected condition or the background response of MoDCs (Figures 1A,B). An anti-MR1 antibody (11) significantly blocked MR1-mediated MAIT cell activation, to an extent comparable to the blockage of cyclosporine that inhibits calcium-mediated signaling cascades in T cell receptor-mediated pathways (35). Statistical analyses support MAIT cell activation upon overnight co-culture and its dependence on MR1-mediated antigen presentation by MoDCs (Figure 1B). Results from three independent assays with a short (5 h) and overnight (15 h) co-culture and anti-MR1 antibody blockage confirmed the activation of MAIT cells and MR1 dependence (Figure 1C). These data demonstrated that MAIT cells were rapidly activated by bacterial-incubated MoDCs within hours in a manner partially dependent on MR1-mediated antigen presentation. However, T cell activation using autologous MoDCs or monocytes is potentially confounded by autoreactivity, as shown with a high CD69 expression of MAIT cells irrelevant to bacterial infection (24). Using bacterial-incubated K562.hMR1 cells, we showed that the human MAIT cell line was also activated in a manner largely blocked by an anti-MR1 antibody and the chemical inhibitor cyclosporine (Figures 1D,E). Three independent assays similarly demonstrated that bacteria-incubated K562.hMR1 rapidly activated MAIT cells with 5 h of co-culture (Figure 1F). Although the MAIT cell line D466F5 was derived from an active tuberculosis patient, its TCR (*TRAV1-2/TRBV6*) was not evidenced being biased to *M. tuberculosis* antigens in this assay (Figure 1) or in other reports (7, 41). Together, these results established a rapid assay of MR1-dependent human MAIT cell activation using K562.hMR1 cells, allowing us to identify surface markers and activation pathways of primary human MAIT cells in the following assays.

### CD69^+^CD26^++^ Labels Activated Human CD8^+^MAIT Cells

**Figure 2:**
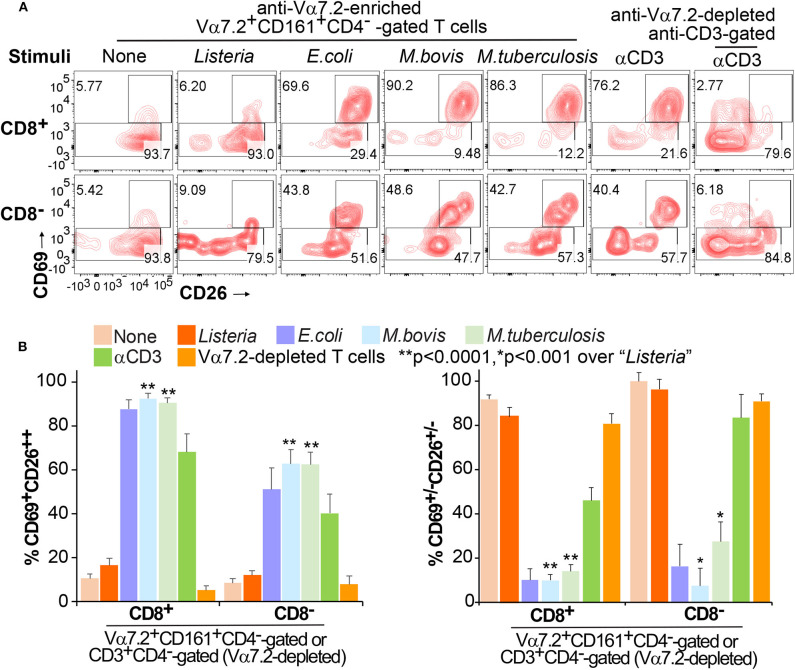
Differentiation of Vα7.2^+^CD161^+^CD4^−^CD8^+^CD69^+^CD26^++^ MAIT cells. Anti-Vα7.2-enriched PBMCs were co-cultured with bacterial-incubated K562.hMR1 cells and anti-Vα7.2-depleted PBMCs were stimulated with anti-CD3 for 15 h. All groups contained an anti-CD28 antibody for co-stimulation. Flow cytometry gated Vα7.2^+^CD161^+^CD4^−^CD8^+^ (or CD8^−^) in anti-Vα7.2-enriched PBMCs and CD3^+^CD4^−^CD8^+^ (or CD8^−^) in anti-Vα7.2-depleted PBMCs. % CD69^+^CD26^++^ MAIT cells (upper) and CD69^+/−^CD26^+/−^ cells (bottom) are annotated. Contour plots show data from one independent assay using PBMCs from one healthy donor **(A)**. Data from four independent assays were plotted with standard errors and analyzed with paired *t*-tests **(B)**.

To date, it remains unclear which surface markers specifically detect and separate activated MAIT cells. The discovery of these markers is crucial for the translational detection of MAIT cells and allows further functional or mechanistic characterization of MAIT cells in bacterial infections and various diseases. Previous studies show that the early T cell activation marker CD69 expresses on stimulated MAIT cells upon bacterial infection; however, CD69 also upregulates in uninfected conditions (8, 12, 24). Moreover, dipeptidyl peptidase (DPP4) named CD26 upregulates on MAIT cells in mycobacterial infection (42). However, CD26 also expresses on MAIT cells in the absence of stimulation (42) and on NK cells (43). Therefore, CD69 or CD26 alone appears unable to separate stimulated MAIT cells from the non-stimulated cells completely. To quantify and define activated MAIT cells, we showed that the co-expression of CD69 and CD26 as a combinatorial marker CD69^+^CD26^++^ separated the activated CD8^+^MAIT cells from the inactivated CD69^+/−^CD26^+/−^ CD8^+^MAIT-enriched cells (Figure 2). The latter population was defined based on the remaining subset in the conditions without bacterium or with the negative control *Listeria* (Figure 2A). In this assay, we co-cultured Vα7.2-enriched human PBMCs with bacterial-incubated K562.hMR1 cells and an anti-CD28 antibody. Upon overnight co-culture, cells were gated at Vα7.2^+^CD161^+^CD4^−^CD8^+^ or Vα7.2^+^CD161^+^CD4^−^CD8^−^ to separate CD69^+^CD26^++^ cells from CD69^+/−^CD26^+/−^ cells (Figure S1), as Vα7.2^+^CD161^+^ gating has been used in multiple studies to detect MAIT cells (8, 42, 44–46), especially bacterial-activated MAIT cells (7, 8, 23). Notably, the percentage of CD69^+^CD26^++^ MAIT cells was dramatically upregulated through co-culture with MR1-expressing cells, which were pre-incubated with *E.coli, M. bovis (BCG)*, and avirulent *M. tuberculosis* (H37Ra) (Figure 2). Moreover, CD69^+^CD26^++^ MAIT cells were clearly separated from CD69^+/−^CD26^+/−^ CD8^+^MAIT-enriched cells. The latter subset remained in non-optimal stimulatory conditions or non-stimulated conditions, such as conditions without bacteria or with non-antigenic *Listeria monocytogenes* (13, 16). Direct stimulation of Vα7.2-enriched cells with anti-CD3/CD28 antibodies as a positive control also upregulated CD69^+^CD26^++^ CD8^+^MAIT cells (Figure 2). Similarly, in the Vα7.2^+^CD161^+^CD4^−^CD8^−^ population, CD69^+^CD26^++^ cells were also upregulated upon various bacterial-stimulations (Figure 2). Moreover, these CD69^+^CD26^++^ MAIT cells nearly disappeared upon anti-MR1 blockage in contrast to the isotype control using an anti-HLA class I antibody (Figure S2), since stimulation using K562.hMR1 cells likely skewed to the induction of MR1-dependent MAIT cell activation. Thus, mycobacterial stimulation consistently upregulates the CD69^+^CD26^++^ CD8^+^MAIT cells dependent on the MR1 molecule, while reversely reduce the CD69^+/−^CD26^+/−^ CD8^+^MAIT-enriched cells (Figure 2B), strongly supporting that CD69^+^CD26^++^ is a specific and sensitive marker to label activated MAITs. These results support that the MR1-dependent MAIT cell activation labeled with a combinatorial marker CD69^+^CD26^++^ can be broadly applied as a promising translational measurement to detect the activated MAIT cells in human.

Recently, the MR1 tetramer loaded with the *E.coli* metabolite 5-amino-6-D-ribitylaminouracil (5-A-RU) (16, 36) was used to stain non-stimulated or metabolite antigen-stimulated MAIT cells from healthy donors or patients (45, 47–50). However, whether the MR1-5-A-RU tetramer also serves as a reliable tool to stain activated MAIT cells in bacterial infection is unknown. To explore this possibility, we co-cultured fresh peripheral blood mononuclear cells (PBMCs) without anti-Vα7.2 and magnetic enrichment with bacterial-incubated K562.hMR1 cells for 15 h. Results showed that the MR1-5-A-RU tetramer was able to stain non-stimulated MAIT cells in PBMCs without bacterial incubation or with *Listeria* incubation. We also showed Vα7.2^+^MR1-5-A-RU^+^ PBMCs were mostly Vα7.2^+^CD161^++^ (Figure S3A), as consistent with previous reports (24, 45, 47, 48). Minimal background staining of the MR1-5-A-RU tetramer in Vα7.2^−^ PBMCs was also similar to that in reports (24, 48). However, MR1-5-A-RU tetramer staining was surprisingly weak or negative for bacterial-stimulated MAIT cells (Figure S3A). Expended gating showed that CD69^+^CD26^++^ cells re-emerged from Vα7.2^+/−^MR1-5-A-RU^+/−^ population (Figure S3B), indicating that mycobacterial-activated MAIT cells downregulated Vα7.2 expression and MR1-5-A-RU staining signals consistent to the report (51). Regardless, activated MAIT cells in the same PBMC samples were still feasibly recognized by Vα7.2 and CD161 antibodies, as eventually labeled by the combinatorial marker of CD69^+^CD26^++^ (Figure S3C) at a similarly high percentage to the anti-Vα7.2-enriched and rested MAIT cells (Figure 2). Therefore, the combinatorial marker CD69^+^CD26^++^ is able to detect activated MAIT cells dependent on MR1-mediated antigen-presentation from Vα7.2^+^CD161^+^ population in bacterial infections. Importantly, CD69^+^CD26^++^ can define human MAIT cells based on MR1 dependence, similar to the definition of CD1-restricted T cells (52, 53) and previous definition of MAIT cells by MR1-dependent activation (7, 8, 10–12).

### CD69^+^CD26^++^ Human CD8^+^MAIT Cells Display a Transcriptome With Multiple Upregulated Activation Markers

**Figure 3:**
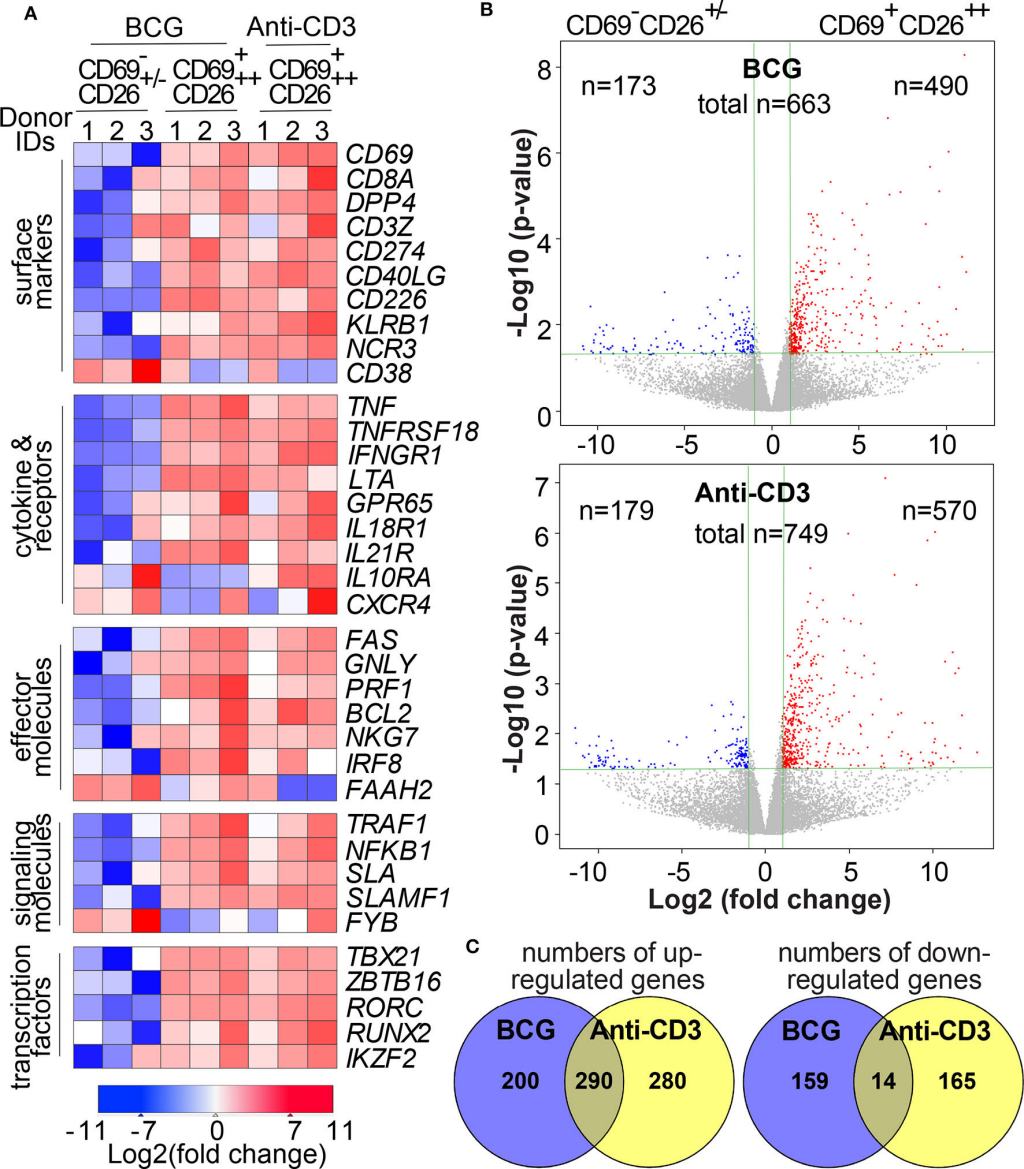
Transcriptomes of Vα7.2^+^CD161^+^CD4^−^CD8^+^CD69^+^CD26^++^ MAIT cells depict multiple activation markers. Upon overnight stimulation using BCG-incubated K562.hMR1 cells or anti-CD3 antibody with anti-CD28 co-stimulation in both conditions, anti-Vα7.2-enriched cells were gated on Vα7.2^+^CD161^+^CD4^−^CD8^+^ cells and further sorted into two subsets, CD69^+^CD26^++^ activated MAITs and CD69^+/−^CD26^+/−^ inactivated MAIT-enriched cells as in Figure 2 and Figure S1. Representative gene expression relevant to MAIT cell activation was compared between BCG-activated MAIT cells and inactivated cells from three healthy donors using anti-CD3 as an activation control. Relative expression levels of these genes are shown in a heatmap with the color scheme representing Log2(fold change) for each gene **(A)**. Volcano plots show up- and down-regulated differentially expressed genes (DEGs) with fold changes (vertical green lines for 2 folds) and *p*-values (horizontal green lines for *p* = 0.05) **(B)**. Venn diagrams show overlapped numbers of DEGs between BCG and anti-CD3 stimulation conditions **(C)**.

Our finding of the activation marker CD69^+^CD26^++^ allows us to separate activated MAIT cells (CD69^+^CD26^++^) unprecedentedly from inactivated MAIT-enriched cells (CD69^+/−^CD26^+/−^) in mycobacterial infection or in disease conditions to elucidate the activation program of Vα7.2^+^CD161^+^CD4^−^CD8^+^ MAIT cells. Recent publications used a MAIT cell antigen (21), anti-CD3 antibody (22), *E.coli* infection (23), and MR1-independent stimulants (22, 23) to determine the transcriptomes of activated MAIT cells. Different from these activation conditions, we would determine the transcriptomic program of abundant human CD8^+^ MAIT cells in response to the initial priming of mycobacterial infection in an MR1-dependent manner. MR1-dependent activation also allow us to specifically define MAIT cells, as the similar definition used for CD1-restricted T cells (52, 53) and in early studies of MAIT cells (7, 8, 10–12). Anti-Vα7.2-enriched human PBMCs (*n* = 3 donors) were co-cultured with BCG-incubated K562.hMR1 cells or stimulated with anti-CD3 antibody for 15 h similarly using anti-CD28 for co-stimulation in all conditions. BCG is the only licensed vaccine against tuberculosis in children and partially protects adults (54). BCG vaccination and *M. tuberculosis* infection enhance MAIT cell frequency in primates, similar to BCG vaccination in humans (55, 56). BCG stimulation also allows us to sort activated primary human MAIT cells through the core facility at an allowed biosafety level for transcriptomic analyses, which inform the MAIT cell activation program in mycobacterial infection. As in Figure S1, we sorted CD69^+^CD26^++^ and CD69^+/−^CD26^+/−^ cells for transcriptomic analyses. We further isolated polyA RNA from sorted cells and performed quality control (27, 28). Then polyA RNA was reversed transcribed, barcoded, and sequenced using the Illumina Hiseq approach as we reported (27, 28). After the determination of gene identities and quantification of intensity counts, we deposited the transcriptomic data to the GEO database (accession number GSE124381) and normalized transcriptomes using the edgeR program. As a result, CD8^+^ MAIT cell transcriptomes detected a total of 14,077 transcripts with minimally 1 count per million (cpm) from at least one out of three donors. Differentially expressed genes (DEGs) were defined with more than 2-fold difference of intensity counts between CD69^+^CD26^++^ activated CD8^+^ MAIT cells and CD69^+/−^CD26^+/−^ inactivated CD8^+^ MAIT-enriched cells. We used a *p*-value (<0.05) to test different gene expression between activated and inactivated Vα7.2^+^CD161^+^CD4^−^CD8^+^ cells from three donors by considering the variability in different donors, *in vitro* bacterial stimulation, T cell activation, cell sorting, and RNA preparation in different assays. To validate the results, we used a heatmap to list multiple clusters of representative genes relevant to the activation of MAIT cells, conventional T cells, and NK cells, as referred below, validating our transcriptomic datasets of the dominantly activated CD69^+^CD26^++^ CD8^+^MAIT cells (Figure 3A). For example, these signature genes encode surface markers *CD69, CD8A, DPP4 (CD26), CD3Z*, and *KLRB1* (CD161); cytokine and receptors *TNF* (TNFα)*, IFNGR1, IL18R1* (IL-18Rα) and *IL21R;* cytolytic effector molecules *GNLY* (granulysin), *PRF1* (perforin) (44), and *FAS* (CD95) (57), signaling molecules *SLA, SLAMF1*, and *NFKB1;* transcription factors *TBX21* (Tbet), *RORC* (RORγt) (46), *IKZF2* (Helios), *RUNX2*, and *ZBTB16* (PLZF) (24, 58) (Figure 3A).

### CD69^+^CD26^++^ Human CD8^+^MAIT Cells Co-express Genes and Share Functional Pathways With Activated T Cells and Innate Immune Cells

**Figure 4:**
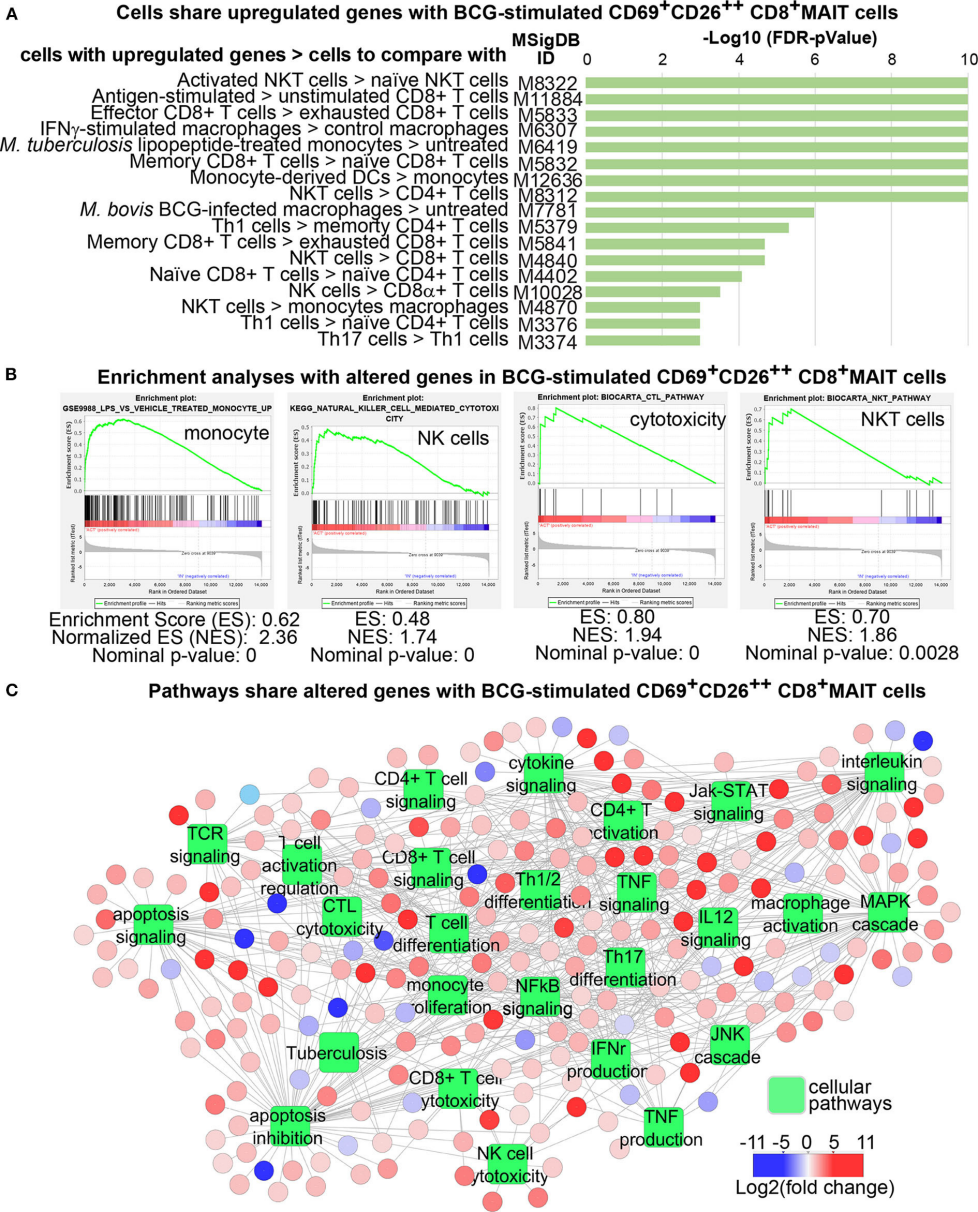
Transcriptomes of Vα7.2^+^CD161^+^CD4^−^CD8^+^CD69^+^CD26^++^ MAIT cells display gene clusters and functional pathways shared with T cells and innate immune cells. Upregulated genes from BCG-stimulated MAIT cells were used to search against the Molecular Signatures Database (MSigDB) and identified significant gene co-expression with more than 3,000 gene datasets (*p* < 0.01). Some representative datasets with various *p*-values are shown **(A)**. Enrichment analyses identified multiple example datasets **(B)**. DEGs from BCG stimulation of MAIT cells significantly overlap with genes in various cellular pathways of activated immune cells (*p* < 0.01) **(C)**.

Although MAIT cell transcriptomes had been shown different from other T cells or between MAIT cell subsets (21, 59), we would address whether mycobacterial infection stimulated an activated transcriptomic program in MAIT cells. Specifically, we determined whether genes differentially expressed between activated MAIT cells vs. inactivated cells. Further, we characterized whether these DEGs in MAIT cells were similar to other adaptive or innate immune cells to support an innate-like activation program associated with anti-mycobacterial functions. To perform these analyses, we started with volcano plots that revealed hundreds of DEGs between activated CD69^+^CD26^++^ CD8^+^MAIT cells and inactivated CD69^+/−^CD26^+/−^ CD8^+^MAIT-enriched cells in BCG and anti-CD3 stimulations, respectively (Figure 3B). The completed lists of up- and down-regulated genes in BCG (*n* = 663 in Table S1) and anti-CD3 (*n* = 749 in Table S2) stimulations were further used for downstream Venn diagram, enrichment, and pathway analyses. Venn diagrams showed large numbers of different and shared genes in BCG and anti-CD3/CD28 stimulation (Figure 3C). In this comparison, 200 upregulated genes (41%) were unique in BCG stimulation and 280 genes (49%) in anti-CD3/CD28 activation. In parallel, 159 downregulated genes (92%) were unique in BCG stimulation and 165 genes (92%) in anti-CD3/CD28 activation. Results supported that BCG stimulation induces a transcriptomic program partially different from that induced by anti-CD3/CD28 antibodies (58), despite that the activated cells were sorted with the same surface markers. To further statistically determine the gene clusters co-expressed with other cell types, we searched the identified BCG-induced DEGs against the gene expression databases in MSigDB (http://software.broadinstitute.org/gsea/msigdb) using the Toppcluster program (37). We identified more than 3,000 of reported gene expression datasets, including conventional CD8^+^ T cells, NKT, NK cells, macrophages, dendritic cells, and other cell types. These T and innate cells co-expressed a large number of genes with BCG-stimulated MAIT cells at a statistically significant level (with a false discovery rate FDR-corrected *p* < 0.01), as shown with multiple representative gene sets (Figure 4A). We also use enrichment analyses to show multiple significantly co-expressed datasets, including NK cells, cytotoxic T cells, NKT cells, and monocytes (Figure 4B). To further annotate functional pathways of activated MAIT cells overlapped with other immune cells, we searched BCG-induced DEGs against various cellular pathways using Toppcluster and constructed a pathway network using the Cytoscape program (http://www.cytoscape.org), based on the cutoff of an FDR-corrected *p*-value at 0.01 (Figure 4C). This network shows that MAIT cells share gene expression in numerous pathways with multiple cell types, including T and NK cell activation, NFκB signaling, MAPK signaling, cytokine productions, and effector functions, etc. Hence, our data reveal that MAIT cell activation shares gene clusters and functional pathways with conventional T cells and innate immune cells.

### CD69^+^CD26^++^ Human CD8^+^MAIT Cells Display a Gene Profile Co-expressed in the T Cell Activation Pathway

**Figure 5:**
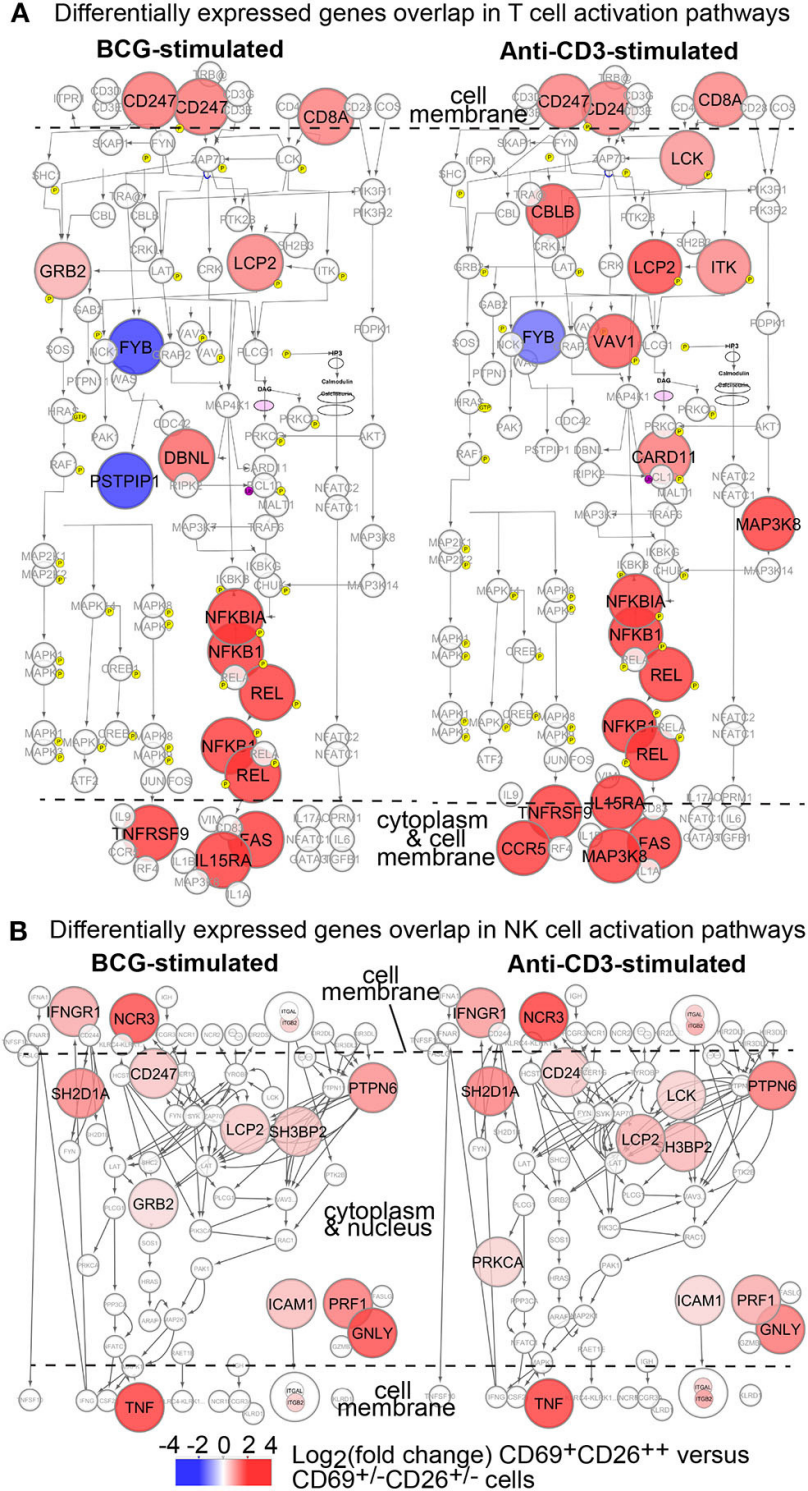
Transcriptomes of Vα7.2^+^CD161^+^CD4^−^CD8^+^CD69^+^CD26^++^ MAIT cells depict overlapped genes in conventional T cell and NK cell activation. DEGs from BCG or anti-CD3 stimulation of MAIT cells are annotated in canonical T cell **(A)** and NK cell **(B)** activation pathways as colored.

From the comprehensive gene profiles shared between MAIT and other immune cells (Figure 4B), it is critical to annotate these shared genes in T cell activation pathways. Therefore, we used Cytoscape to obtain a map of canonical T cell activation pathways (60) to annotate BCG-altered genes. The upregulated genes included *CD3Z* (CD247), *CD8A*, and *LCP2*, downstream transcription factors *NFKB1A, NFKB1*, and *REL*, which together supported a TCR-mediated NFκB pathway (60) (Figure 5A). Other genes include *PTPN6* (SHP1) important for TCR signaling (61), *TNF, BCL2, TRAF1, IL15Ra, TNFRSF9* (4-1BB), and *FAS* for regulating effector function. Moreover, MAIT cells stimulated with anti-CD3/CD28 antibodies induced additional signaling molecules, including *ITK, LCK*, and *VAV1* (Figure 5A). Anti-CD3/CD28 antibodies appear capable of stimulating both NFκB- and MAP3 Kinase-mediated signaling pathways (62, 63), likely promoting more comprehensive singling transduction in comparison to BCG stimulation. These data demonstrate that activated MAIT cells share common signature genes in canonical activation pathways with conventional T cells.

### CD69^+^CD26^++^ Human CD8^+^MAIT Cells Display a Gene Profile Co-expressed in the NK Cell Activation Pathway

It is known that MAIT cells express NK cell receptors CD161 and NCR3, as in our findings (Figures 2, 3). To understand whether activated MAIT cells also share gene signatures in activated NK cells, we similarly obtained canonical NK cell activation pathways through database search and comprehensively annotated BCG-altered MAIT cell genes (Figure 5B). Co-expressed genes included those encoding cell surface molecules, an IFNγ receptor (*IFNGR1*) for cytokine stimulation and NK cell chemotaxis (64), and a natural cytotoxicity triggering receptor *NCR3 (NKp30*) for CD3ξ molecule (*CD247*) interaction and NK cell differentiation (65). Co-expressed genes also encoded multiple signaling molecules, such as *SH2D1A* (SAP) and *PTPN6* (SHP1), which are critical in both NK and T cells (61, 66). Ultimately, the upregulation of effector molecules, such as cytokine TNF (67, 68), cytolytic molecule perforin (PRF1) (57), and adhesion molecule ICAM1, enhance the protection against infection, killing of bacterial-infected cells, and cell migration to infected tissues. Therefore, activated CD8^+^MAIT cells also share signature gene expression in the NK cell activation pathway (Figure 5B).

### CD69 and CD26 Label Pro-inflammatory Responses of Human CD8^+^MAIT Cells in *M. tuberculosis* Infection

**Figure 6:**
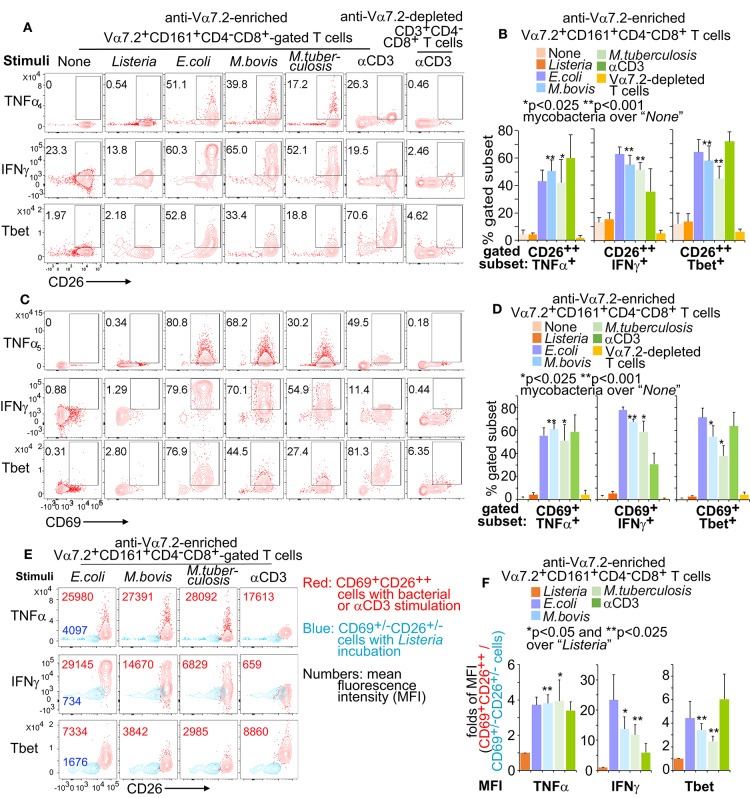
Pro-inflammatory cytokines from activated Vα7.2^+^CD161^+^CD4^−^CD8^+^ cells. Anti-Vα7.2-enriched PBMCs were similarly activated with bacterial-incubated K562.hMR1 cells or anti-CD3 with anti-CD28 presented in all conditions as in Figure 2. Flow cytometry gated Vα7.2^+^CD161^+^CD4^−^CD8^+^ cells from anti-Vα7.2-enriched PBMCs and gated CD3^+^CD4^−^CD8^+^ cells from anti-Vα7.2-depleted PBMCs. Contour plots show % positive cells on gated Vα7.2^+^CD161^+^CD4^−^CD8^+^CD26^++^ in one independent assay with PBMCs from one healthy donor **(A)**. Data from four independent assays were plotted with standard errors and analyzed with the paired *t*-test **(B)**. Vα7.2^+^CD161^+^CD4^−^CD8^+^CD69^+^ MAIT cells are also plotted **(C)** and statistically tested **(D)**. Vα7.2^+^CD161^+^CD4^−^CD8^+^CD69^+^CD26^++^-gated activated MAIT cells in bacterial or anti-CD3 stimulation (Red) were further compared with Vα7.2^+^CD161^+^CD4^−^CD8^+^CD69^+/−^CD26^+/−^ inactivated cells in *Listeria* incubation (Blue) by displaying the contour plots and mean fluorescence intensity (MFI) for noted molecules. Data from one independent assay using a blood sample from a healthy donor were shown **(E)**. Fold difference of MFI between gated activated and inactivated cells were plotted with standard errors for data from four independent assays. Paired *t*-tests were performed to test the difference between mycobacterial stimulation and listeria incubation **(F)**.

Pro-inflammatory responses have been demonstrated as critical effector functions against mycobacterial infection (3, 69, 70). Our transcriptomic analyses suggest an enhanced pro-inflammatory response of MAIT cells upon BCG stimulation, including an enhanced expression of *TNF, TBX21* (encoding the transcription factor Tbet), and *IFNGR1*. TNFα bears a prominent early anti-tuberculosis function, which was shown in mouse studies (68) and also supported by severe tuberculosis in human autoimmune diseases with anti-TNFα therapies (67). IFNγ is another critical cytokine in controlling the infection of *M. tuberculosis* (71). To determine whether primary human MAIT cells are quickly activated to produce anti-mycobacterial pro-inflammatory cytokines, we similarly stimulated MAIT cells with bacterial-incubated K562.hMR1 cells (Figure 2) and validated activated MAIT cells by gating on Vα7.2^+^CD161^+^CD4^−^CD8^+^CD69^+^CD26^++^ cells (Figure S1). Results showed the upregulated frequency of CD26^++^TNFα^+^ (Figures 6A,B) and CD69^+^TNFα^+^ (Figures 6C,D) MAIT cells in Vα7.2^+^CD161^+^CD4^−^CD8^+^ upon bacterial and anti-CD3/CD28 stimulation. Further gating on CD69^+^CD26^++^ from bacterial-stimulated conditions annotated high TNFα expression subset in comparing to un-stimulated CD69^+/−^CD26^+/−^ subset in Listeria-incubated condition (Figures 6E,F). Consistently, we demonstrated similar results with IFNγ (Figure 6), which display a high degree of enhancement. The expression of transcription factor Tbet is also upregulated (Figure 6), similar to the enhanced *TBX21* expression in the transcriptome of BCG-stimulated MAIT cells. Together, anti-mycobacterial pro-inflammatory responses are enhanced in response to *E.coli* and mycobacterial infections.

### Stimulated Human MAIT Cells Enhanced Cytolytic Functions and Inhibited Mycobacterial Growth

**Figure 7:**
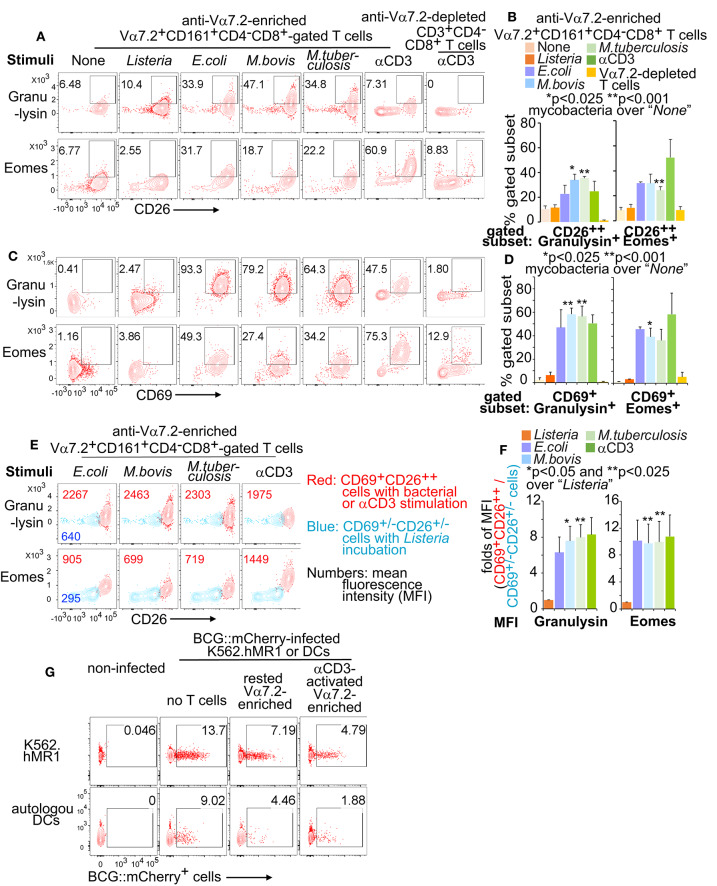
Cytolytic molecules from activated Vα7.2^+^CD161^+^CD4^−^CD8^+^ cells. Anti-Vα7.2-enriched PBMCs were activated and gated as described in Figure 6. Contour plots show % positive cells of cytolytic molecules on gated Vα7.2^+^CD161^+^CD4^−^CD8^+^CD26^++^ in one independent assay with PBMCs from one healthy donor **(A)**. Data from three independent assays were plotted with standard errors and analyzed with paired *t*-tests **(B)**. Vα7.2^+^CD161^+^CD4^−^CD8^+^CD69^+^ MAIT cells are also plotted **(C)** and statistically tested **(D)**. Vα7.2^+^CD161^+^CD4^−^CD8^+^CD69^+^CD26^++^ activated MAIT cells (Red) were compared with Vα7.2^+^CD161^+^CD4^−^CD8^+^CD69^+/−^CD26^+/−^ inactivated cells (Blue) with contour plots and mean fluorescence intensity (MFI) for noted molecules as in Figure 6. Data from one independent assay were shown **(E)**. Fold difference of MFI between activated and inactivated cells from four independent assays were plotted and statistically tested with paired *t*-tests **(F)**. The BCG.mCherry-incubated MoDCs and K562.hMR1 cells were washed for 4 times and co-cultured with rested or anti-CD3/CD28-activated MAIT-enriched cells for 18 h. Data show remained % mCherry^+^ cells from an assay using a blood sample of a healthy donor and three independent assays show similar results **(G)**.

Human MAIT cells express cytolytic molecules at an un-stimulated condition or upon the activation of fixed *E.coli* (24, 44, 72). However, it is unclear whether mycobacterial infection also stimulates MAIT cells to express cytolytic molecules and display a protective function. Overnight incubation of MAIT cells with BCG- or H37Ra-stimulated K562.hMR1 cells enhanced the % CD26^++^ and CD69^+^ MAIT cells expressing granulysin and Eomesodermin (Eomes) (Figures 7A–D). The gated CD69^+^CD26^++^ MAIT cells also upregulated the fluorescent staining intensity of granulysin and Eomes (Figures 7E,F). Results support that bacterial stimulation further upregulates cytolytic molecules over the basal level of expression. To determine whether both pro-inflammatory cytokines and cytolytic function together play a role to inhibit mycobacterial growth, we co-cultured the unstimulated or anti-CD3-stimulated Vα7.2-enriched cells with K562.hMR1 cells or human MoDCs pre-infected with mCherry-labeled BCG (39). Results demonstrated that either rested or pre-stimulated MAIT-enriched cells inhibited the growth of the mCherry-labeled BCG detected with flow cytometry (Figure 7G). Since pro-inflammatory cytokines and cytolytic functions of conventional T cells have been shown to fight mycobacterial infections (67, 68), our data support human MAIT cells similarly inhibit mycobacterial growth as in a previous report with mouse MAIT cells (18).

## Discussion

The hypothesis of “innate-like T cells” was perhaps raised from the early observation of MAIT cell activation that depends on the interaction of a monomorphic MR1 protein, an antigen, and a semi-invariant TCR (9–14, 73–75). This conserved genetics and physical interaction in the MR1-antigen-TCR complex had likely provided a primordial mechanism that preserved innate features in MAIT cell activation and preceded the peptide antigen presentation (9–14), as similarly exemplified in CD1-restricted T cell activation (53, 75–77). In this study, we defined a combinatorial marker to label rapidly activated MAIT cells for the characterization of transcriptomic programs and effector functions against mycobacterial infections. Results reveal gene expression profiles of mycobacterial-activated MAIT cells share with activated CD8^+^ T cells and NK cells (Figures 3–5). Effector responses, including the enhanced production of pro-inflammatory cytokines (TNFα and IFNγ) and the cytolytic molecule (granulysin) (Figures 3, 4, 6, 7), and the inhibition of mycobacterial growth (Figure 7), are also similar to activated CD8^+^ T cells and NK cells (78). This unique innate-like activation program of MAIT cells is expected to provide a niche of early protection against *M. tuberculosis* infection, by potentially bridging innate (79) and adaptive immune responses (4, 5).

An activation marker is crucial for labeling and defining MAIT cells to further estimate MAIT effector functions in mycobacterial infections and other diseases. Previous reports mainly focused on detecting MAIT cells from blood samples using Vα7.2, CD161, or MR1 tetramer in humans and mice (7, 8, 24, 45, 47, 48). Nonetheless, it is challenging to isolate activated MAIT cells from the inactivated in bacterially infected conditions, due to the lack of activation-related surface markers for a complete separation and the potential downregulation of TCR expression that minimizing the signals of anti-Vα7.2 and tetramer staining on activated MAIT cells (Figure S3). Our results showed the TCR downregulation on mycobacterial-stimulated MAIT cells similar to that on activated conventional T cells (80), NKT cells (81), and MAIT cells (51), potentially contributing to a negative feedback regulation of TCR-mediated T cell responses (82). To overcome the difficulty of detecting activated MAIT cells, we used the combinatorial marker CD69^+^CD26^++^ to label a high percentage of Vα7.2^+^CD161^++^CD4^−^CD8^+^ cells at an MR1-dependent activation condition (Figure 2) as blocked by the anti-MR1 antibody (Figure S2). This combinatorial marker is highly translational to specifically detect activated vs. inactivated MAIT subsets from individual tuberculosis patients without using controls of other bacterial-infected patients. Likewise, CD69^+^CD26^++^ can be determined whether it is a candidate marker to label activated MAIT cells in *M. tuberculosis*-infected animal models. Particularly, the animal models are highly demanded to replicate abundant MAIT cell frequency, CD8^+^ MAIT cell responses, and *M. tuberculosis*-infected lung pathology in humans. As the major MAIT cell subset in humans (7, 8, 55), CD8^+^ MAIT cells can be stimulated to generate a higher percentage of CD69^+^CD26^++^ cells than CD4^−^CD8^−^ MAIT cells in mycobacterial stimulation (*p* < 0.01). Interestingly, CD26 expression on CD8^+^ T cells has been associated with pro-inflammatory responses as a potential co-stimulatory molecule (83) and cytolytic responses as CD26 co-localizes with granulysin, perforin, and granzymes intracellularly (84). Thus, the high expression of CD26 and the differentiation of a CD69^+^CD26^++^ subset can perhaps label the activated CD8^+^ MAIT cells that produce pro-inflammatory and cytolytic molecules upon *M. tuberculosis* stimulation as in this study. Indeed, rapidly activated MAIT cells in *M. tuberculosis* stimulation consist of a higher percentage of CD8^+^ MAIT cells but a lower percentage of the CD4^−^CD8^−^ subset than inactivated MAIT cells (Figure 2), consistent to the responses of CD8^+^ and CD4^−^CD8^−^ MAIT cells in *E.coli* stimulation (49). Moreover, the differentiation kinetics and effector functions of CD8^+^ and CD4^−^CD8^−^ MAIT cells in chronic *M. tuberculosis* infection require comprehensive investigations, as extended *E.coli* stimulation can decrease CD8^+^ MAIT cell frequency (49). To understand the early activation program of CD8^+^MAIT cells in mycobacterial stimulation, we used a heatmap to show the expression of multiple signature genes from these CD69^+^CD26^++^ CD8^+^MAIT cells, including surface markers, cytokines, cytolytic molecules, and transcription factors. These molecules have been mostly reported in activated MAIT, other T cells, and NK cells (8, 24, 42–46, 57, 58), validating the activation of CD69^+^CD26^++^ MAIT cells in mycobacterial infection (Figure 3A). Our results are also complementary to the findings that MAIT cells can be activated in a manner independent of MR1-mediated antigen presentation. For example, the stimulation with cytokine IL-18 also upregulates the expression of CD69 and IFNγ, independent on anti-MR1 blockage at viral-infected conditions (85, 86). The cellular context and molecular stimulants for MR1-dependent and independent MAIT cell activation are expected to be complementary and similarly important for inducing anti-microbial responses.

Recently, MAIT cell transcriptomes were compared with other T cells (59), associated with tissue repair function (21, 22), and coordination of immune responses (23). However, it remains unclear whether MAIT cells rapidly activated in pathogenic bacterial infections show an innate-like activation program to mediate protective responses. To understand the innateness of MAIT cell activation in mycobacterial infection beyond the conserved molecular interaction of MR1-antigen-TCR complex (12–14, 73, 74), we determined MAIT cell transcriptomes between activated MAIT cells Vα7.2^+^CD161^+^CD4^−^CD8^+^CD69^+^CD26^++^ and inactivated MAIT-enriched cells Vα7.2^+^CD161^+^CD4^−^CD8^+^CD69^+/−^CD26^+/−^ in BCG stimulation. Our findings provide multiple lines of evidence to support the innate-like activation program of MAIT cells in mycobacterial stimulation. First, we compared DEGs (*n* = 663) from BCG-stimulated MAIT cells with various innate or adaptive immune cells and revealed thousands of datasets with co-expressed genes at high statistical significance (Figures 4A,B). Second, DEGs of CD69^+^CD26^++^ MAIT cells also significantly overlap with various pathways typically shown in activated CD8^+^ T cells, NK cells, and NKT cells (Figure 4C). These overlapped pathways broadly involve activation, differentiation, proliferation, cytotoxicity, cytokine production (e.g., IFNγ, TNF), apoptosis, and intracellular signaling, such as NFκB-, MAPK-, Jak-STAT-mediated signaling, in conventional T cells and innate immune cells (Figure 4C). Third, DEGs of activated MAIT cells overlap with both T cells and NK cells in their activation pathways (Figure 5). For example, CD3ζ (CD247) is important in coupling the antigen recognition of T cells and the CD16 surface ligation of NK cells to intracellular signaling pathways (87, 88). CD8α is critical in cytotoxic T cell and NK cell responses (89). NFκB contributes to the pro-inflammatory response and cell survival of T cells (90, 91), and receptor-mediated signaling transduction in innate immune cells (92). Upregulated genes also include multiple TNF superfamily factors, which usually interact with the NFκB-mediated pathway in conventional T cells and innate immune cells (93). Shared with NK cell activation pathways as well, genes encoding cell surface receptors *IFNGR1* and *NCR3* (NKp30) are crucial in other innate-like T cell populations, such as γδT cells (94, 95). Together, co-expression, enrichment, overlapped functional pathways, and shared genes in activation pathways between activated MAIT cells and other innate or adaptive immune cells strongly support the innateness of MAIT cell activation.

We expect this innate-like activation program and the combinatorial activation marker can be translated to understand early anti-mycobacterial MAIT cell immune responses in humans and animal models. First, our observation supports that rapid MAIT cell responses are potentially important in early protection against *M. tuberculosis* infection, relevant to previous findings of the conserved tri-molecular interaction in humans (73, 74) and the early inhibition of mycobacterial growth in mice (8, 18). BCG vaccination and *M. tuberculosis* infections are suggested to transiently enhance MAIT cell frequency in rhesus macaques (56), similar to transient MAIT cell responses to BCG vaccination in humans (55). This early MAIT cell response *in vivo* is consistent to the innate-like activation program of MAIT cells in this study, although the kinetics, trends, and distributions of MAIT cells or their activation in human *M. tuberculosis* infections requires further investigations (7, 96–98). Second, we observed that both BCG and avirulent H37Ra similarly activated primary human MAIT cells. However, virulent *M. tuberculosis* is usually less efficient in stimulating conventional T cells (99, 100), due to its virulence in causing necrosis and reducing antigen presentation capability of infected cells (101, 102), except for T cell responses specific to the unique antigens of virulent *M. tuberculosis* (103). For MAIT cells, BCG and *M. tuberculosis* have been suggested to comparably enhance peripheral MAIT cell frequency in the early stage of vaccination or infection of rhesus macaques (56), supporting a rapid MAIT cell activation. However, different from BCG vaccination, chronic lung pathology in *M. tuberculosis* infection may contribute to the redistribution of MAIT cells from blood to lung tissues and pleural space (42, 96, 97). Third, we showed that CD69^+^CD26^++^ CD8^+^MAIT cells upregulated the gene and protein expression of multiple pro-inflammatory and cytolytic molecules, such as TNFα, IFNγ, granulysin, perforin, and corresponding master transcription factors Tbet and Eomes, upon overnight mycobacterial stimulation. Anti-mycobacterial responses have been usually reflected by the production of pro-inflammatory cytokines from CD4^+^ and CD8^+^ T cells, and innate cells (3, 69, 70), together with the cytolytic function of CD8^+^ T cells (4). Relevant to the enhanced production of various pro-inflammatory cytokines and cytolytic molecules of early activated MAIT cells, co-culture of MAIT cells with mycobacterial-incubated K562.hMR1 cells and MoDCs inhibits the growth of mCherry-labeled BCG. These data support the potential protection of human MAIT cells against early mycobacterial infection, similar to the inhibition of BCG growth by mouse MAIT cells (18). Moreover, rapidly stimulated effector functions of activated CD8^+^MAIT cells likely facilitate the kicking in of adaptive immune responses to provide a full set of immune protection, representing a unique niche in anti-mycobacterial immune responses.

## Data Availability Statement

The datasets presented in this study can be found in online repositories. The names of the repository/repositories and accession number(s) can be found below: https://www.ncbi.nlm.nih.gov/genbank/ with an accession number GSE124381.

## Ethics Statement

Blood samples free of detectable infectious or non-infectious diseases were obtained from healthy donors with written informed consent at the Hoxworth Blood Center in the University of Cincinnati. De-identified blood samples were processed according to the protocols approved by the Institutional Review Board of the University of Cincinnati.

## Author Contributions

All authors: reviewed the manuscript. MS and SZ: perform assays, initial data analyses, and interpretation. LN: initial transcriptomic data analyses. DL: generation of MAIT cell lines. XZ: RNA sample preparation and sequencing. SH: study design, data analyses, and manuscript writing.

## Conflict of Interest

The authors declare that the research was conducted in the absence of any commercial or financial relationships that could be construed as a potential conflict of interest.

## Abbreviations

MAIT: mucosal-associated invariant T
MHC: major histocompatibility complex
MR1: MHC-related protein 1
CD: cluster of differentiation
NK: natural killer
NKT: natural killer T cells
TNFα: tumor necrosis factor α
IFNγ: interferon γ
TCR: T cell receptor
HLA: human leukocyte antigens
PBMCs: peripheral blood mononuclear cells
MoDCs: monocyte-derived dendritic cells
LPS: lipopolysaccharide
BCG: Bacille Calmette-Guerin
*M. bovis*: *Mycobacterium bovis*
*M. tuberculosis*: *Mycobacterium tuberculosis*
*E. coli*: *Escherichia coli*
*L. monocytogenes*: *Listeria monocytogenes*
ELISPOT: enzyme-linked immunospot
Vα7.2: variable segment 7.2 of the T cell receptor alpha chain
APC/Cy7: Allophycocyanin/Cyanine7
PE: phycoerythrin
DEGs: differentially expressed genes
FDR: false discovery rate
Tbet: T-box transcription factor
Eomes: omesodermin.
