# Supplementary material for: Mucosal-Associated Invariant T Cells Develop an Innate-Like Transcriptomic Program in Anti-mycobacterial Responses
Source: Front Immunol. 2020 Jun 9;11:1136. doi: 10.3389/fimmu.2020.01136 (PMC7295940; doi:10.3389/fimmu.2020.01136)
Supplement: Supplementary file 1 [file Data_Sheet_1.pdf]

Fig. S1 Sharma et al.

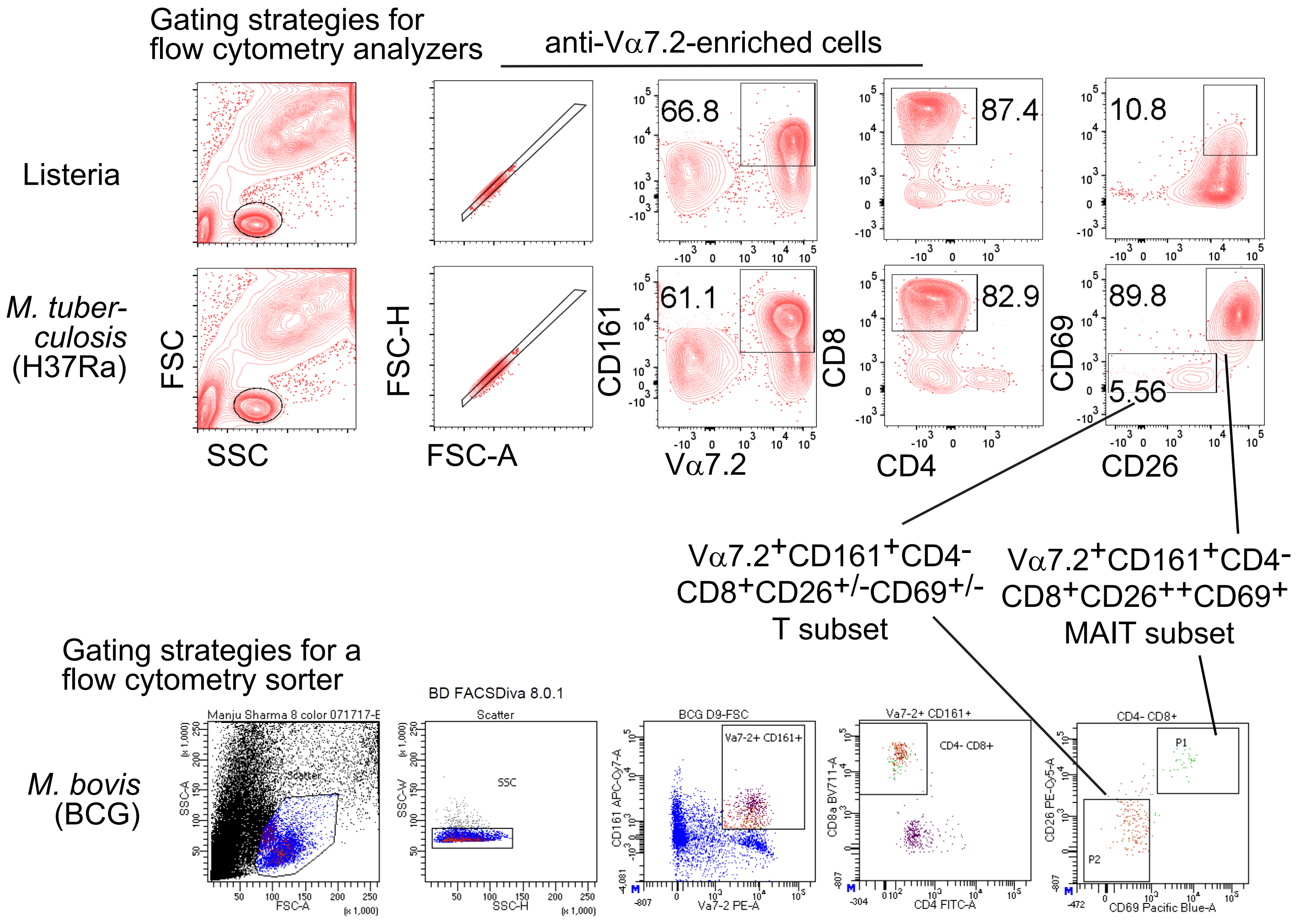

Fig. S1 Gating of Vα7.2^{+}CD161^{+} T cells in mycobacterial stimulation. Human PBMCs were sorted with anti-Vα7.2 antibody and co-cultured with *M. tuberculosis*-incubated K562.hMR1 cells as described in Fig. 2. We further gate co-cultured cells on Vα7.2^{+}CD161^{+}CD4^{-}CD8^{+} for CD69^{+}CD26^{++} and CD69$^{+/-}$CD26$^{+/-}$ cell analysis and sorting.

Fig. S2 Sharma et al.

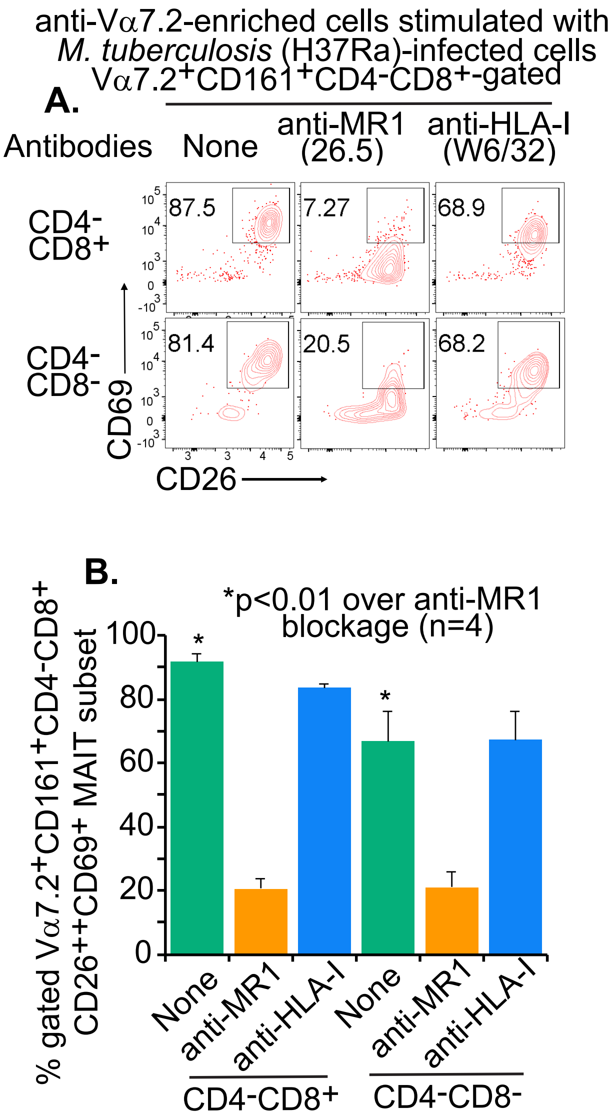

Fig. S2 Blockade of MAIT cell responses in mycobacterial stimulation. Human PBMCs were sorted with anti-Vα7.2 antibody and co-cultured with *M. tuberculosis*-incubated K562.hMR1 cells as described in Fig. 2. Upon co-culture for 15 hours, we gate on Vα7.2^{+}CD161^{+}CD4^{-}CD8^{+} cells to determine % CD26^{++}CD69^{+} labeled as activated MAIT cells. Anti-MR1 antibody (clone 26.5, IgG2a) and anti-HLA-A, B, C (HLA-I clone W6/32) with the same isotype were added at the cell co-culture. Contour plots from one donor are shown (**A**) and % CD26^{++}CD69^{+} are plotted using blood samples of four donors (**B**).

Fig. S3 Sharma et al.

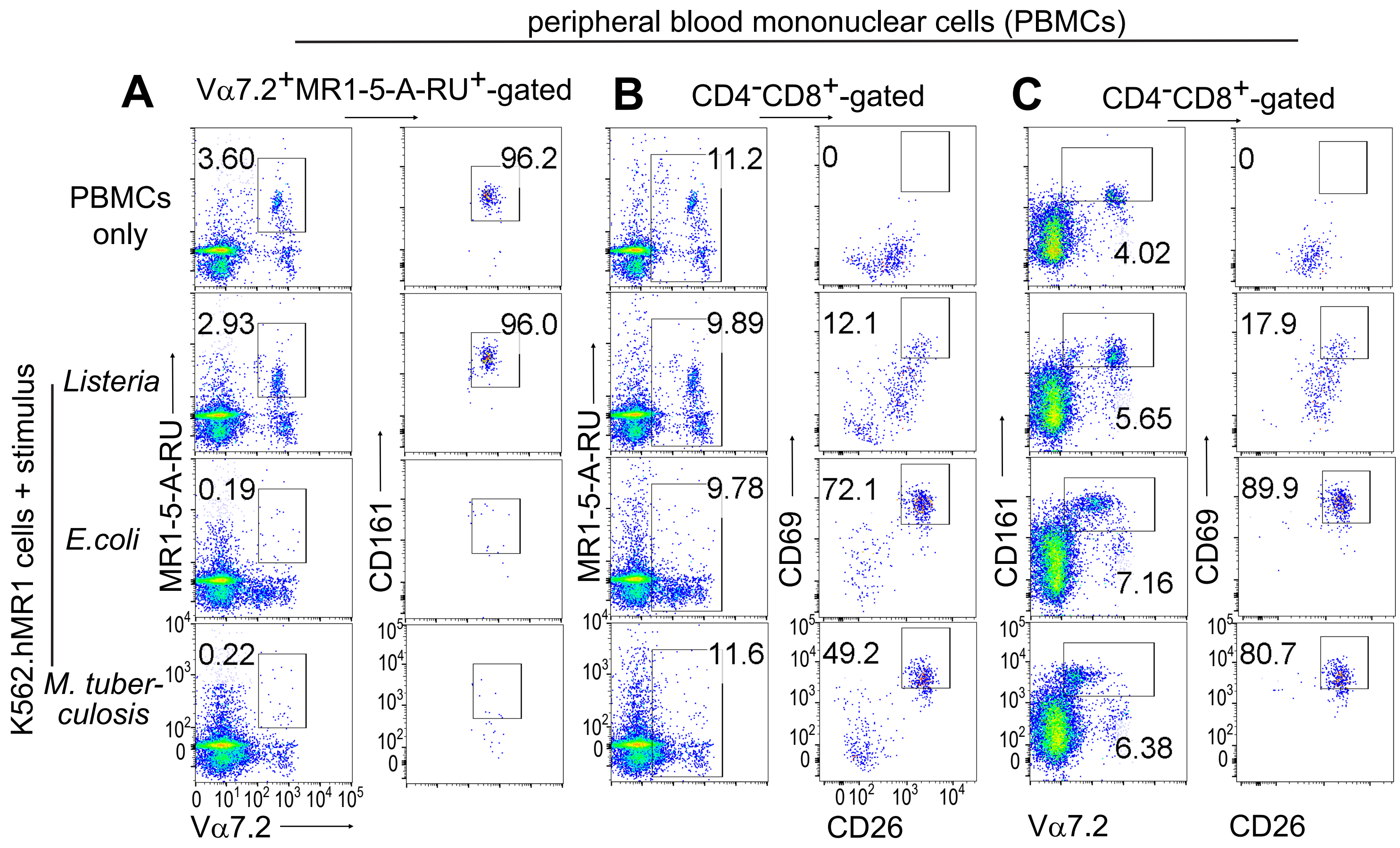

Fig. S3 Mycobacterial-stimulated MAITs are detectable with the anti-Vα7.2 antibody and show minimal staining with MR1-5-A-RU tetramer. PBMCs from a healthy donor were incubated with bacterial-stimulated K562.hMR1 cells for 15 hours in comparison to PBMCs along. PBMCs were stained and gated with MR1-5-A-RU tetramer (tet) and the anti-Vα7.2 antibody on Vα7.2^{+}tet^{+} cells **(A)** or Vα7.2$^{+/-}$tet$^{+/-}$ cells (**B**). Gating with Vα7.2^{+}CD161^{+} as in Fig. 2 and Fig. S1 is compared **(C).** Similar results were obtained with blood samples from two healthy donors.

Supplemental Table 1: MAIT cell gene expression altered in BCG stimulation.

ID	Gene names	Log2 (fold change)	Fold change	P Value
1	ZBED2	11.14	2262.002	6.09E-04
2	CCL20	11.09	2175.518	5.47E-09
3	AOC2	10.95	1975.157	3.83E-02
4	THEM5	10.94	1965.820	2.70E-04
5	CD226	10.60	1547.767	4.28E-03
6	TNFRSF18	10.15	1133.767	9.76E-07
7	SNORD81	10.03	1047.594	1.72E-02
8	ELOVL4	9.74	853.828	3.21E-02
9	SCARNA17	9.69	827.678	1.74E-02
10	LACC1	9.60	773.767	1.33E-03
11	NFKBID	9.59	772.725	7.96E-06
12	METTL25	9.55	752.011	3.23E-02
13	GRID2IP	9.52	735.879	8.61E-03
14	OAS1	9.29	625.781	2.20E-02
15	IL1RL1	9.20	589.634	4.93E-02
16	HOPX	9.09	544.663	2.14E-06
17	BLK	9.06	533.035	1.23E-02
18	MED12L	8.99	507.189	1.86E-02
19	HLA-DMB	8.89	473.726	4.68E-02
20	MIR205HG	8.84	458.312	3.87E-02
21	CCL4	8.82	453.176	4.66E-05
22	NAP1L2	8.69	413.384	3.34E-02
23	MIR142	8.65	402.574	9.99E-03
24	ZNF416	8.53	370.377	4.86E-02
25	ZNF583	8.46	352.742	3.22E-03
26	FSD1	8.03	261.240	2.87E-02
27	LOC100132111	7.67	203.351	5.06E-03
28	ROBO3	7.47	177.435	1.06E-02
29	OVCA2	7.40	169.246	2.70E-02
30	RORC	7.40	168.801	8.47E-06
31	FAM159A	7.36	164.102	4.43E-02
32	TSSK4	7.30	157.666	3.29E-02
33	CD79B	7.22	148.679	3.86E-02
34	NAPSA	6.97	125.569	3.75E-02
35	BCL2A1	6.86	115.943	3.18E-03
36	LTA	6.79	110.454	9.44E-06
37	TNF	6.68	102.720	1.58E-07
38	ADGRG5	6.58	95.779	3.96E-03
39	PLGRKT	6.07	66.985	4.10E-02
40	CCDC134	6.02	64.834	2.15E-02
41	CXCR6	6.02	64.795	1.04E-02
42	UBASH3B	6.00	63.843	7.22E-03
43	PSMB8-AS1	5.90	59.878	6.51E-03
44	PDGFA	5.86	57.928	3.62E-03
45	VDR	5.72	52.542	2.43E-03

46	CCL3	5.67	50.811	3.35E-03
47	EGR2	5.66	50.567	8.08E-04
48	IL21R	5.57	47.404	4.07E-04
49	DUSP2	5.56	47.268	2.42E-04
50	CD40LG	5.56	47.144	9.13E-04
51	IL23A	5.44	43.482	1.56E-05
52	COLQ	5.35	40.794	1.65E-03
53	B3GALT2	5.22	37.208	6.17E-03
54	IRF8	5.20	36.672	7.10E-03
55	TNFRSF9	5.20	36.632	8.31E-05
56	TIAM1	5.19	36.603	2.92E-03
57	PTGER2	5.17	36.056	1.80E-02
58	IL4I1	5.15	35.414	2.08E-03
59	GRASP	5.14	35.371	5.62E-03
60	COL5A3	5.04	32.956	3.20E-03
61	LIF	5.02	32.401	2.79E-02
62	LST1	4.87	29.225	3.96E-03
63	SLC4A10	4.78	27.443	1.83E-04
64	KLRG1	4.76	27.081	4.89E-05
65	DUSP5	4.74	26.665	2.95E-03
66	NCR3	4.65	25.087	3.77E-05
67	SLAMF1	4.61	24.477	8.07E-04
68	GNLY	4.60	24.231	1.52E-02
69	C17orf96	4.57	23.706	5.64E-03
70	USP30	4.32	20.002	4.08E-03
71	JOSD2	4.24	18.861	9.81E-03
72	ZBTB16	4.19	18.244	1.32E-02
73	LONRF1	4.06	16.640	4.32E-02
74	CD82	4.05	16.560	2.59E-05
75	JAKMIP1	4.04	16.495	1.51E-03
76	TNFSF8	3.92	15.142	5.94E-03
77	IL15RA	3.91	15.080	2.54E-02
78	FOSL2	3.87	14.593	3.91E-04
79	METRNL	3.70	13.010	6.38E-03
80	ARNTL	3.67	12.709	3.53E-02
81	HIC1	3.60	12.094	1.02E-04
82	ADAM19	3.55	11.689	2.43E-02
83	AGFG2	3.54	11.609	5.53E-03
84	NINJ1	3.51	11.373	1.51E-03
85	PRF1	3.41	10.599	4.81E-06
86	SEMA7A	3.36	10.296	1.37E-02
87	HIVEP3	3.31	9.938	2.51E-02
88	IL18RAP	3.30	9.826	2.04E-02
89	BATF	3.22	9.292	4.44E-02
90	NR4A1	3.19	9.125	1.57E-03
91	RGCC	3.17	9.022	3.34E-02
92	TNFSF14	3.16	8.957	2.03E-03

93	FAS	3.15	8.902	9.20E-03
94	CDK6	3.12	8.696	2.86E-02
95	CD69	3.12	8.672	5.80E-03
96	STX11	3.12	8.670	4.86E-03
97	SIPA1L1	3.11	8.652	1.86E-02
98	PHLDA1	3.11	8.639	3.71E-04
99	KLRB1	3.10	8.589	9.35E-03
100	ADAM12	3.10	8.559	4.21E-02
101	MDFIC	3.09	8.541	1.86E-04
102	UPP1	3.09	8.522	2.92E-02
103	PYROXD1	3.09	8.520	1.01E-02
104	PRDM1	3.06	8.353	5.69E-04
105	SH2D1A	3.05	8.274	3.39E-02
106	TBX21	2.95	7.754	3.94E-03
107	TESK1	2.94	7.669	1.52E-02
108	SLA	2.92	7.551	1.67E-03
109	RBPJ	2.91	7.506	8.06E-06
110	SPHK2	2.90	7.481	2.09E-02
111	C10orf54	2.90	7.472	6.09E-04
112	MYO1G	2.90	7.471	6.49E-04
113	PLK3	2.88	7.340	1.65E-04
114	NR4A2	2.87	7.333	2.60E-02
115	CD274	2.85	7.187	2.42E-02
116	PTPN6	2.84	7.142	8.24E-05
117	LEMD3	2.84	7.140	3.38E-02
118	MAF	2.81	6.994	3.24E-04
119	LAG3	2.80	6.968	7.47E-03
120	APOBEC3G	2.79	6.917	4.07E-04
121	DPP4	2.79	6.903	5.93E-04
122	SIPA1L2	2.73	6.633	3.17E-02
123	IKZF2	2.67	6.352	5.05E-03
124	IL2RB	2.65	6.284	5.41E-05
125	DOK2	2.64	6.233	1.85E-03
126	TRAF4	2.62	6.163	1.87E-02
127	PBX4	2.62	6.159	2.98E-02
128	NKG7	2.62	6.157	6.59E-03
129	NFKB1	2.62	6.154	2.83E-05
130	GFI1	2.61	6.119	9.11E-04
131	BTBD11	2.61	6.090	1.14E-02
132	KDM6B	2.60	6.077	1.68E-04
133	IL18R1	2.59	6.038	8.90E-03
134	TRAF1	2.59	6.030	5.08E-05
135	SLC25A22	2.58	5.966	1.33E-02
136	M6PR	2.56	5.895	4.03E-02
137	RGS3	2.53	5.765	3.46E-03
138	FKBP11	2.52	5.749	8.96E-04
139	PLEK	2.50	5.652	1.23E-02

140	GPR171	2.49	5.627	2.31E-04
141	ITGA1	2.43	5.378	5.72E-04
142	SH2D2A	2.40	5.270	3.89E-05
143	BHLHE40	2.39	5.226	5.66E-04
144	REL	2.38	5.198	7.18E-05
145	SRGN	2.37	5.156	3.19E-05
146	MAFK	2.34	5.059	9.13E-03
147	ADAP1	2.33	5.011	1.35E-02
148	LTB	2.32	5.008	3.06E-03
149	CISH	2.32	4.977	2.43E-02
150	TAGAP	2.31	4.955	1.26E-02
151	ACSL6	2.30	4.916	1.86E-03
152	BIRC3	2.30	4.916	4.73E-05
153	NFKBIA	2.28	4.854	2.66E-05
154	BACH2	2.28	4.844	1.98E-03
155	GPR65	2.27	4.833	5.72E-04
156	GNG2	2.27	4.824	3.38E-03
157	SNHG15	2.19	4.576	1.47E-02
158	PTGER4	2.18	4.543	2.09E-02
159	LOC284454	2.17	4.499	2.69E-02
160	IER3	2.16	4.473	1.33E-03
161	ZFP36L1	2.15	4.453	2.65E-05
162	CELF2	2.14	4.409	1.59E-04
163	PLAGL2	2.13	4.372	3.65E-03
164	IFNGR1	2.11	4.331	3.40E-04
165	SLC41A1	2.10	4.293	2.75E-02
166	RORA	2.09	4.260	4.51E-04
167	LPAR2	2.08	4.217	4.12E-02
168	RARA	2.08	4.215	3.41E-03
169	NAMPT	2.07	4.195	1.10E-03
170	PTPN22	2.03	4.088	1.86E-03
171	SLC16A3	2.02	4.042	9.68E-03
172	CD2	2.01	4.021	8.50E-04
173	NCS1	2.00	4.000	3.56E-03
174	NPTN	1.99	3.982	9.32E-03
175	RAB8B	1.97	3.915	4.18E-04
176	NCOA7	1.96	3.902	7.46E-04
177	SDC4	1.96	3.898	4.44E-03
178	FOXJ2	1.96	3.879	1.58E-02
179	GADD45B	1.93	3.807	7.27E-03
180	SCRN1	1.92	3.781	3.35E-02
181	SLC39A14	1.91	3.752	1.47E-02
182	NFAT5	1.90	3.733	2.37E-03
183	RNH1	1.88	3.690	7.51E-03
184	DOT1L	1.88	3.683	2.21E-03
185	PNO1	1.87	3.665	5.37E-03
186	PIM3	1.87	3.648	1.14E-03

187	AMPD2	1.87	3.644	4.73E-02
188	CREM	1.87	3.643	2.72E-03
189	FURIN	1.86	3.630	5.07E-04
190	JAML	1.86	3.621	3.95E-02
191	IL4R	1.84	3.587	6.15E-04
192	BCL2L1	1.84	3.577	2.00E-03
193	SERPINB9	1.82	3.526	2.40E-03
194	GIT1	1.81	3.503	1.17E-02
195	RASSF5	1.81	3.496	1.02E-02
196	NEK7	1.80	3.479	3.32E-03
197	FEZ1	1.78	3.443	6.71E-03
198	ARFGAP3	1.78	3.439	6.99E-03
199	CD44	1.78	3.431	1.16E-03
200	SP4	1.77	3.417	4.18E-02
201	PHACTR2	1.76	3.378	3.67E-03
202	LPXN	1.73	3.326	1.03E-02
203	KIF21B	1.73	3.320	6.51E-03
204	CHPF	1.73	3.306	1.43E-02
205	DDIT4	1.72	3.300	3.50E-03
206	BTG2	1.72	3.293	3.45E-02
207	GOLT1B	1.72	3.288	9.98E-03
208	CAMK2D	1.71	3.280	1.58E-02
209	SEC24D	1.71	3.266	8.76E-03
210	ITGB7	1.70	3.254	3.20E-02
211	CMTM6	1.69	3.218	2.35E-02
212	MAP2K3	1.69	3.216	7.55E-03
213	DUSP16	1.68	3.204	1.12E-02
214	NFKBIZ	1.68	3.201	3.02E-03
215	IL32	1.68	3.194	4.59E-04
216	HYOU1	1.67	3.181	2.14E-02
217	FBXO41	1.66	3.168	4.86E-03
218	PDE4D	1.65	3.144	1.11E-02
219	ARHGEF2	1.64	3.123	3.65E-02
220	PELO	1.64	3.117	1.90E-02
221	ID2	1.64	3.116	1.22E-02
222	B4GALT5	1.64	3.110	1.95E-02
223	ARHGAP30	1.64	3.108	6.03E-04
224	FYCO1	1.63	3.105	2.33E-02
225	LFNG	1.63	3.101	6.76E-03
226	TNFAIP3	1.63	3.100	3.00E-03
227	ICAM1	1.63	3.095	3.97E-03
228	BCL2	1.63	3.094	9.21E-03
229	IGF2R	1.61	3.062	1.56E-03
230	ATP6V0E1	1.61	3.061	3.56E-02
231	UFM1	1.61	3.057	1.55E-02
232	NFKB2	1.61	3.056	6.49E-03
233	CCDC124	1.61	3.050	3.14E-02

234	ECE1	1.60	3.034	1.04E-02
235	PSME2	1.60	3.032	1.24E-03
236	C9orf69	1.59	3.013	2.24E-02
237	AGAP2	1.59	3.001	1.47E-02
238	PROSC	1.58	3.000	4.86E-02
239	GRPEL1	1.58	2.993	3.50E-02
240	ANXA6	1.58	2.990	9.93E-04
241	APBB1IP	1.57	2.977	3.61E-02
242	DBNL	1.57	2.970	2.87E-02
243	SLC25A32	1.56	2.957	1.19E-02
244	CCDC43	1.56	2.940	3.39E-02
245	UBE2M	1.55	2.937	4.48E-02
246	DNPEP	1.55	2.929	1.18E-02
247	ITCH	1.54	2.901	2.70E-02
248	PRNP	1.54	2.899	5.05E-03
249	CCND2	1.52	2.866	4.43E-03
250	FNBP1	1.51	2.857	3.10E-03
251	SHMT2	1.51	2.855	3.54E-02
252	MDM2	1.51	2.853	3.99E-03
253	STAT4	1.50	2.838	4.25E-03
254	TRIM25	1.50	2.834	7.49E-03
255	PPP1R10	1.50	2.830	1.27E-02
256	WARS	1.50	2.824	5.81E-03
257	BTG1	1.49	2.817	1.92E-03
258	TRAK2	1.48	2.785	2.00E-02
259	CERK	1.47	2.775	6.44E-03
260	AKR1A1	1.47	2.769	1.92E-02
261	RFLNB	1.46	2.755	4.07E-02
262	SLC7A1	1.46	2.753	6.19E-03
263	ADGRE5	1.45	2.740	3.33E-03
264	NUS1	1.45	2.735	2.50E-03
265	PGGHG	1.45	2.735	3.65E-04
266	TNFRSF1B	1.45	2.730	1.18E-03
267	RPS6KA1	1.45	2.725	1.10E-02
268	SPOP	1.45	2.725	7.49E-03
269	FMNL1	1.44	2.718	1.64E-03
270	SH3BP2	1.44	2.718	8.81E-03
271	AEBP2	1.44	2.717	4.59E-02
272	TTPAL	1.44	2.714	2.27E-02
273	CAPN15	1.44	2.713	1.09E-02
274	MAP2K4	1.44	2.708	3.70E-02
275	ZNFX1	1.44	2.707	3.60E-02
276	KIF3B	1.43	2.699	5.15E-03
277	ZC3H12A	1.43	2.697	1.10E-02
278	GOLGA8A	1.43	2.691	2.52E-02
279	TXNL4A	1.43	2.687	3.69E-02
280	SARS2	1.42	2.679	3.32E-02

281	TTC39C	1.42	2.677	9.91E-03
282	PKM	1.42	2.675	6.20E-04
283	CHRAC1	1.41	2.661	3.49E-02
284	CD320	1.41	2.661	4.97E-02
285	MYC	1.41	2.657	5.64E-03
286	RNF19A	1.40	2.643	1.75E-03
287	RPL22L1	1.40	2.640	3.70E-02
288	CD247	1.40	2.638	2.37E-02
289	VPS4B	1.40	2.631	1.89E-02
290	LTBP4	1.39	2.618	7.18E-03
291	TESPA1	1.39	2.618	2.68E-02
292	GPX1	1.38	2.608	3.87E-02
293	CBX6	1.38	2.603	4.78E-03
294	FOXO1	1.38	2.599	4.74E-02
295	CLPTM1	1.38	2.599	3.87E-02
296	FHOD1	1.38	2.596	2.14E-02
297	PRKCH	1.37	2.592	5.97E-03
298	NGDN	1.37	2.581	3.36E-02
299	SH3KBP1	1.37	2.580	2.57E-02
300	RPS6KA3	1.36	2.570	1.37E-03
301	GTF3C1	1.36	2.568	4.88E-02
302	LTBP3	1.36	2.565	3.64E-02
303	CYTIP	1.36	2.564	3.89E-02
304	MAPK13	1.36	2.559	3.39E-02
305	C12orf49	1.35	2.557	4.23E-02
306	TNFAIP8	1.35	2.555	4.45E-03
307	CPD	1.35	2.555	2.60E-02
308	FOXP1	1.35	2.553	8.56E-03
309	GNL1	1.35	2.550	1.67E-02
310	WDR46	1.35	2.544	2.33E-02
311	PEAK1	1.34	2.532	1.81E-02
312	JARID2	1.34	2.530	2.00E-02
313	MCRIP1	1.34	2.528	4.59E-02
314	MSN	1.34	2.527	1.31E-02
315	SLC7A5	1.33	2.517	6.45E-03
316	PMM2	1.33	2.511	2.34E-02
317	TRMT1	1.33	2.510	6.17E-03
318	MYBBP1A	1.33	2.507	1.62E-02
319	SEL1L	1.32	2.500	1.90E-02
320	GEMIN4	1.32	2.497	3.17E-02
321	CD6	1.32	2.496	6.23E-03
322	UTP20	1.32	2.494	9.25E-03
323	RELB	1.32	2.489	2.69E-02
324	NRIP1	1.31	2.485	4.91E-02
325	RBMS1	1.31	2.484	4.15E-02
326	CD8A	1.31	2.480	1.21E-02
327	SRPRB	1.31	2.476	1.57E-02

328	EMB	1.30	2.460	1.59E-02
329	SELENOT	1.29	2.450	9.64E-03
330	EFHD2	1.29	2.439	1.21E-02
331	TINF2	1.29	2.438	3.71E-02
332	NAA20	1.27	2.419	3.42E-02
333	LCP2	1.27	2.418	2.15E-02
334	DNMT3A	1.26	2.400	1.03E-02
335	C21orf91	1.26	2.400	4.53E-02
336	ELOVL1	1.26	2.391	1.76E-02
337	ITGB2	1.25	2.382	9.71E-03
338	RABGGTB	1.25	2.378	3.71E-02
339	IL12RB1	1.25	2.374	2.62E-02
340	STRAP	1.25	2.373	4.12E-02
341	DUSP4	1.24	2.369	1.58E-02
342	SNRPB	1.24	2.364	4.50E-02
343	CASP4	1.24	2.361	3.38E-02
344	RASSF2	1.23	2.339	3.48E-02
345	RBM27	1.23	2.338	4.76E-02
346	SRPRA	1.22	2.337	4.97E-03
347	TMEM30A	1.22	2.334	3.15E-03
348	FUBP1	1.21	2.313	2.70E-03
349	GALNT1	1.21	2.311	4.15E-02
350	AGL	1.20	2.302	4.66E-02
351	GPATCH4	1.20	2.291	4.77E-02
352	MOB3A	1.19	2.288	2.60E-02
353	KMT2B	1.19	2.278	1.66E-02
354	FKBP4	1.18	2.273	2.95E-02
355	PAG1	1.18	2.265	3.87E-03
356	KIF5C	1.18	2.265	4.75E-02
357	FTSJ3	1.17	2.252	2.20E-02
358	MYDGF	1.17	2.250	1.06E-02
359	EBNA1BP2	1.16	2.241	4.25E-02
360	NOP58	1.16	2.235	1.40E-02
361	GNPDA1	1.16	2.235	3.46E-02
362	SLC35A4	1.16	2.232	2.74E-02
363	CLUH	1.16	2.232	3.18E-02
364	MFSD14A	1.16	2.230	2.78E-02
365	LUZP1	1.16	2.230	2.51E-02
366	PPRC1	1.15	2.222	1.85E-02
367	ZMIZ2	1.15	2.220	3.95E-02
368	HERPUD1	1.15	2.219	4.86E-02
369	CTDNEP1	1.14	2.206	2.93E-02
370	CAD	1.14	2.196	9.89E-03
371	SNHG16	1.12	2.180	2.99E-02
372	PRMT1	1.12	2.178	1.28E-02
373	IMPAD1	1.12	2.175	4.88E-02
374	SDF4	1.12	2.174	4.58E-02

375	LCP1	1.12	2.172	1.93E-02
376	WDR74	1.12	2.172	2.34E-02
377	MED24	1.12	2.170	1.51E-02
378	PPM1M	1.12	2.169	3.76E-02
379	TSPYL2	1.11	2.157	3.12E-02
380	NFKBIB	1.11	2.156	3.23E-02
381	LSP1	1.11	2.155	1.89E-02
382	GLS	1.10	2.150	2.44E-02
383	CARS	1.10	2.144	3.55E-02
384	TMEM167A	1.10	2.137	8.79E-03
385	STAT5A	1.09	2.131	4.97E-02
386	IVNS1ABP	1.09	2.127	1.17E-02
387	B4GALT1	1.08	2.120	1.26E-02
388	PURB	1.08	2.115	1.53E-02
389	PREX1	1.08	2.113	2.90E-02
390	SEC14L1	1.08	2.113	2.18E-02
391	PUM3	1.08	2.112	4.32E-02
392	NDFIP1	1.07	2.101	1.10E-02
393	NR1H2	1.07	2.099	2.80E-02
394	MTDH	1.07	2.098	3.13E-02
395	SZRD1	1.07	2.098	2.03E-02
396	MANF	1.07	2.097	3.57E-02
397	CCND3	1.07	2.097	5.09E-03
398	C14orf166	1.06	2.090	4.52E-02
399	LYST	1.06	2.084	3.62E-02
400	XBP1	1.06	2.079	1.14E-02
401	NUP62	1.06	2.078	2.26E-02
402	IL2RG	1.05	2.077	4.29E-02
403	CARMIL2	1.05	2.076	1.51E-02
404	WDR55	1.05	2.071	3.16E-02
405	PSME1	1.05	2.066	1.83E-02
406	HIF1A	1.05	2.065	9.68E-03
407	PRR14	1.04	2.061	2.13E-02
408	DDX21	1.04	2.053	4.51E-02
409	DNAJC3	1.03	2.049	2.88E-02
410	TGFBR2	1.03	2.043	1.30E-02
411	HMGN4	1.03	2.043	3.83E-02
412	RPN1	1.03	2.036	3.03E-02
413	VCP	1.03	2.035	1.27E-02
414	PATL1	1.02	2.033	2.94E-02
415	ACSL4	1.02	2.023	3.01E-02
416	UBE3C	1.02	2.022	4.40E-02
417	TNFRSF25	1.00	1.998	2.08E-02
418	RBM8A	1.00	1.995	1.22E-02
419	SATB1	0.99	1.989	2.23E-02
420	OSBPL3	0.99	1.986	2.80E-02
421	HARS	0.99	1.982	2.19E-02

422	NADK	0.99	1.981	4.56E-02
423	NCLN	0.98	1.978	2.60E-02
424	AZIN1	0.98	1.978	2.95E-02
425	CES2	0.98	1.977	4.44E-02
426	EFCAB14	0.98	1.967	3.25E-02
427	DNAJC14	0.98	1.967	4.58E-02
428	RPL36AL	0.98	1.966	2.01E-02
429	KRI1	0.96	1.952	2.80E-02
430	STK4	0.96	1.951	4.51E-02
431	DNAJA1	0.96	1.942	2.40E-02
432	RHOG	0.96	1.940	3.95E-02
433	PFKP	0.96	1.939	3.45E-02
434	EIF5A	0.95	1.937	1.93E-02
435	PITPNB	0.95	1.937	2.08E-02
436	NCBP2	0.95	1.931	1.91E-02
437	ARPC2	0.95	1.930	8.97E-03
438	PITPNM1	0.94	1.923	3.10E-02
439	HIPK2	0.94	1.919	2.54E-02
440	UBE2J1	0.93	1.909	3.26E-02
441	RANGAP1	0.91	1.880	4.88E-02
442	ZC3HAV1	0.91	1.877	8.32E-03
443	TNIP1	0.91	1.874	1.63E-02
444	MAFG	0.91	1.874	4.49E-02
445	BZW1	0.90	1.869	2.84E-02
446	SEC23B	0.90	1.868	2.80E-02
447	ARHGDIA	0.90	1.865	1.45E-02
448	SPEN	0.90	1.861	2.06E-02
449	TACC1	0.90	1.860	3.76E-02
450	SSR4	0.89	1.857	4.71E-02
451	IPO4	0.89	1.856	3.13E-02
452	NACC1	0.89	1.855	3.38E-02
453	DANCR	0.89	1.855	4.82E-02
454	CDC37	0.88	1.847	4.09E-02
455	PRDX1	0.88	1.842	3.51E-02
456	CORO1A	0.88	1.835	2.07E-02
457	RBM17	0.87	1.824	4.32E-02
458	DDOST	0.86	1.819	2.72E-02
459	SLC3A2	0.86	1.818	2.55E-02
460	PIM2	0.86	1.815	2.31E-02
461	TLN1	0.86	1.812	2.46E-02
462	ADIPOR2	0.85	1.808	3.35E-02
463	SMG7	0.85	1.808	4.86E-02
464	LITAF	0.85	1.806	3.18E-02
465	SYNCRIP	0.85	1.799	4.47E-02
466	ATP13A3	0.84	1.792	4.07E-02
467	PDIA3	0.84	1.792	1.61E-02
468	CDV3	0.84	1.785	1.99E-02

469	TNRC18	0.83	1.776	3.89E-02
470	MYH9	0.81	1.755	7.25E-03
471	DDX54	0.81	1.752	4.59E-02
472	WDR43	0.81	1.751	3.88E-02
473	HSPH1	0.80	1.745	3.62E-02
474	USP7	0.80	1.740	3.05E-02
475	LMAN1	0.79	1.733	1.34E-02
476	GRB2	0.79	1.729	3.70E-02
477	ABCC1	0.78	1.720	3.03E-02
478	ACTR3	0.78	1.712	4.88E-02
479	STAT3	0.76	1.690	4.54E-02
480	TXNRD1	0.75	1.677	3.14E-02
481	IPO5	0.73	1.659	4.85E-02
482	EIF3D	0.72	1.648	3.86E-02
483	SRCAP	0.71	1.636	3.11E-02
484	SSR2	0.71	1.633	4.90E-02
485	KPNA4	0.70	1.627	4.29E-02
486	TAP2	0.69	1.614	4.04E-02
487	HDLBP	0.68	1.602	4.74E-02
488	PFN1	0.68	1.601	3.47E-02
489	TNPO1	0.67	1.593	4.59E-02
490	MLLT6	0.66	1.576	4.71E-02
491	MALAT1	-0.67	0.627	4.88E-02
492	TMSB4X	-0.69	0.621	4.31E-02
493	SF3B1	-0.72	0.609	1.64E-02
494	SARAF	-0.75	0.594	3.83E-02
495	ZMYM2	-0.75	0.594	4.34E-02
496	USP48	-0.81	0.571	2.55E-02
497	PNISR	-0.85	0.556	1.19E-02
498	STAT1	-0.85	0.554	3.30E-02
499	DCAF8	-0.86	0.550	3.58E-02
500	NARF	-0.87	0.546	4.79E-02
501	AHNAK	-0.88	0.545	2.31E-02
502	SUN2	-0.90	0.536	2.23E-02
503	ZC3H7A	-0.91	0.530	4.62E-02
504	KDM5A	-0.93	0.525	4.46E-02
505	MRPS27	-0.94	0.522	3.21E-02
506	RIF1	-0.95	0.517	4.26E-02
507	UTRN	-0.95	0.516	4.33E-02
508	ORC2	-1.00	0.501	4.99E-02
509	KIAA1551	-1.01	0.497	9.97E-03
510	LRCH4	-1.04	0.487	2.00E-02
511	AHSA2	-1.04	0.487	3.54E-02
512	ZNF451	-1.06	0.481	4.66E-02
513	TAOK1	-1.06	0.480	2.24E-02
514	KAT6A	-1.07	0.476	3.17E-02
515	ARHGEF6	-1.07	0.476	4.28E-02

516	ARHGEF1	-1.09	0.470	1.31E-02
517	PLEKHA2	-1.10	0.468	3.93E-02
518	HERC1	-1.10	0.468	1.82E-02
519	CPOX	-1.10	0.466	3.05E-02
520	ACER3	-1.10	0.465	4.53E-02
521	CLSPN	-1.11	0.465	4.00E-02
522	ABI2	-1.11	0.464	4.72E-02
523	42984	-1.12	0.460	4.71E-02
524	HECA	-1.13	0.456	1.87E-02
525	SENP7	-1.16	0.446	3.79E-02
526	TBC1D10C	-1.21	0.431	3.03E-02
527	TYMS	-1.21	0.431	4.39E-02
528	SYNE1	-1.22	0.429	3.31E-02
529	TRANK1	-1.22	0.428	3.73E-02
530	TBC1D2B	-1.23	0.426	1.54E-02
531	EVL	-1.28	0.412	5.05E-03
532	UBA7	-1.29	0.409	2.54E-02
533	ARHGAP45	-1.29	0.409	7.06E-03
534	ST6GAL1	-1.33	0.398	3.34E-02
535	IL7R	-1.33	0.398	1.75E-02
536	STAT2	-1.34	0.396	2.38E-02
537	SORL1	-1.34	0.394	2.37E-02
538	PARP12	-1.35	0.393	3.63E-02
539	FAM102A	-1.35	0.392	3.41E-02
540	ARSA	-1.36	0.390	2.66E-02
541	TRIM22	-1.40	0.378	4.07E-03
542	TNRC6B	-1.41	0.376	8.77E-03
543	YPEL3	-1.41	0.375	3.77E-02
544	CBX7	-1.43	0.372	4.69E-02
545	RASA3	-1.46	0.364	1.18E-02
546	PATJ	-1.48	0.359	2.47E-02
547	CASP8AP2	-1.49	0.357	1.55E-02
548	NLRC3	-1.49	0.355	9.77E-03
549	IL16	-1.51	0.352	4.81E-02
550	GABARAPL1	-1.52	0.349	1.29E-02
551	RBL2	-1.53	0.346	1.78E-02
552	CLK3	-1.56	0.339	4.42E-02
553	PDXDC2P	-1.56	0.338	2.62E-02
554	NELL2	-1.57	0.338	7.88E-03
555	SAT1	-1.57	0.336	3.78E-02
556	MVP	-1.59	0.332	4.12E-02
557	IL10RA	-1.61	0.327	2.31E-02
558	COA1	-1.63	0.323	3.34E-02
559	PIGO	-1.64	0.320	3.92E-02
560	CXCR4	-1.65	0.318	2.82E-02
561	SLFN5	-1.66	0.316	4.29E-02
562	BTN3A1	-1.71	0.306	5.14E-03

563	CALCOCO1	-1.77	0.293	2.80E-02
564	GIMAP7	-1.78	0.290	1.76E-02
565	PBXIP1	-1.80	0.288	4.13E-02
566	KLHL24	-1.81	0.285	1.19E-02
567	FBXL20	-1.81	0.285	2.45E-02
568	PIDD1	-1.83	0.281	3.93E-02
569	SYNE2	-1.84	0.279	2.59E-04
570	MX1	-1.84	0.279	1.32E-02
571	KCTD7	-1.92	0.265	2.38E-02
572	ATM	-1.92	0.264	6.25E-04
573	CYTH4	-1.95	0.259	2.90E-02
574	ACSS1	-2.00	0.251	3.79E-02
575	PIGX	-2.00	0.250	2.71E-02
576	ZNF337	-2.01	0.248	1.34E-02
577	APBB1	-2.05	0.242	2.83E-02
578	LINC00342	-2.11	0.231	9.87E-03
579	PSTPIP1	-2.15	0.226	1.52E-02
580	ACAP1	-2.17	0.222	7.75E-04
581	CD84	-2.21	0.216	6.37E-03
582	PASK	-2.21	0.216	5.69E-03
583	SELPLG	-2.31	0.201	1.97E-02
584	HAPLN3	-2.34	0.197	5.74E-03
585	DDX58	-2.43	0.186	3.69E-03
586	FYB	-2.51	0.175	2.50E-04
587	PDE3B	-2.59	0.166	8.15E-04
588	MYLK3	-2.63	0.161	2.10E-02
589	TBC1D16	-2.65	0.160	4.73E-02
590	GPR155	-2.72	0.152	3.25E-02
591	TMOD2	-2.73	0.151	3.11E-02
592	MFSD13A	-2.81	0.143	4.01E-02
593	LINC00476	-2.82	0.142	1.53E-02
594	CREBRF	-2.93	0.131	2.68E-03
595	LINC00861	-3.18	0.110	2.09E-02
596	ARID5B	-3.24	0.106	7.22E-03
597	ZNF99	-3.25	0.105	4.99E-02
598	CEP290	-3.30	0.101	4.07E-02
599	EPSTI1	-3.38	0.096	1.82E-02
600	SGSH	-3.42	0.093	4.49E-02
601	ABLIM1	-3.51	0.088	5.64E-03
602	SESN3	-3.64	0.080	4.04E-03
603	MIAT	-3.67	0.079	2.85E-04
604	PIK3IP1	-3.74	0.075	1.08E-02
605	ABTB1	-3.75	0.074	4.67E-02
606	PCDH12	-3.92	0.066	4.84E-02
607	PROM2	-3.98	0.063	4.54E-02
608	ZC3H12D	-4.07	0.059	3.15E-02
609	MYO15B	-4.12	0.057	1.21E-02

610	GSDMA	-4.45	0.046	3.91E-02
611	LOC101928131	-5.04	0.030	3.51E-02
612	KANTR	-5.19	0.027	3.05E-02
613	SLC16A4	-5.21	0.027	2.81E-02
614	OVGP1	-5.23	0.027	2.75E-02
615	ADCY10P1	-5.36	0.024	4.46E-02
616	P2RX7	-5.42	0.023	2.45E-02
617	ZFP3	-5.57	0.021	2.71E-02
618	LY9	-5.62	0.020	7.87E-03
619	LOC101926935	-5.70	0.019	3.66E-02
620	SIRPG	-5.86	0.017	2.66E-02
621	ZNF80	-5.99	0.016	3.72E-02
622	CPEB3	-6.08	0.015	1.49E-02
623	FCGBP	-6.11	0.014	1.80E-03
624	MYOM1	-6.53	0.011	3.39E-02
625	NECTIN3	-6.55	0.011	3.29E-02
626	MDGA1	-6.61	0.010	2.08E-02
627	LOC339059	-6.74	0.009	4.82E-02
628	C5orf17	-6.80	0.009	4.79E-02
629	MMP24	-6.90	0.008	4.98E-02
630	ASRGL1	-6.92	0.008	3.04E-02
631	IQCH	-7.00	0.008	4.98E-02
632	CD38	-7.05	0.008	4.00E-02
633	SDHAF4	-7.51	0.005	4.62E-02
634	DCAF4L1	-7.79	0.005	2.96E-02
635	TCTN1	-7.81	0.004	3.19E-02
636	DIP2C	-7.89	0.004	2.15E-02
637	KLHL7-AS1	-7.91	0.004	3.74E-02
638	LINC00689	-8.29	0.003	4.27E-02
639	LRRC37A6P	-8.37	0.003	4.06E-02
640	TRIQK	-8.47	0.003	3.41E-02
641	ADAMTSL1	-8.49	0.003	3.95E-02
642	MATN1-AS1	-9.09	0.002	1.23E-02
643	ZSWIM5	-9.09	0.002	4.70E-02
644	TRIM67	-9.10	0.002	3.19E-02
645	HIST1H3A	-9.20	0.002	3.49E-02
646	VNN2	-9.32	0.002	3.82E-02
647	CABYR	-9.41	0.001	3.55E-02
648	ABCD2	-9.47	0.001	1.04E-02
649	CFAP43	-9.61	0.001	3.29E-02
650	GNAO1	-9.62	0.001	1.77E-02
651	DNAJC15	-9.64	0.001	1.42E-02
652	SCN1B	-9.78	0.001	4.56E-02
653	BRE-AS1	-9.79	0.001	4.40E-02
654	LOC100506985	-9.79	0.001	2.76E-02
655	CEMP1	-9.85	0.001	1.18E-02
656	RAB37	-10.11	0.001	1.72E-02

657	FGGY	-10.17	0.001	2.65E-02
658	MAGEE1	-10.20	0.001	2.30E-02
659	AFDN-AS1	-10.23	0.001	4.45E-02
660	DLGAP4-AS1	-10.34	0.001	4.06E-02
661	FAAH2	-10.36	0.001	3.79E-03
662	SIRT4	-10.75	0.001	3.85E-02
663	TMEM71	-10.81	0.001	3.23E-02

Supplemental Table 2: MAIT cell gene expression altered in anti-CD3 stimulation.

ID	Gene names	Log2 (fold change)	Fold change	P Value
1	PLEKHA7	12.70	6649.098	2.39E-02
2	BLK	11.77	3486.204	4.41E-03
3	ZNF416	11.70	3326.017	1.94E-02
4	ZBED2	11.59	3086.144	5.06E-04
5	LACC1	11.38	2664.131	6.24E-04
6	RGAG4	11.29	2501.566	3.62E-02
7	THEM5	11.23	2397.940	2.39E-04
8	SARM1	10.93	1951.565	3.00E-02
9	CXorf65	10.78	1763.773	1.73E-02
10	CXCR6	10.75	1723.155	3.66E-04
11	PDE1B	10.52	1467.104	3.15E-02
12	MT1X	10.24	1205.184	2.50E-02
13	FSD1	10.15	1137.876	1.29E-02
14	TNFRSF18	10.15	1132.369	9.82E-07
15	TNFRSF18	10.11	1106.188	1.48E-02
16	ARMCX4	10.08	1081.673	4.04E-02
17	AMZ2P1	10.04	1051.985	3.81E-02
18	MIR142	10.02	1041.553	6.08E-03
19	MIR205HG	10.02	1037.653	2.35E-02
20	NTSR1	9.92	971.953	4.35E-02
21	PLCXD2	9.83	910.336	3.78E-02
22	HOPX	9.73	846.388	1.40E-06
23	CD226	9.59	770.075	6.19E-03
24	USP18	9.54	745.768	4.81E-02
25	HLA-DMB	9.48	711.909	3.53E-02
26	NLGN3	9.31	636.567	2.39E-02
27	NFKBID	9.04	525.265	1.11E-05
28	TCEAL3	8.74	427.111	3.10E-02
29	NAP1L2	8.69	414.110	3.31E-02
30	LOC100996286	8.66	404.975	4.94E-02
31	CCDC154	8.60	386.699	3.97E-02
32	CD79B	8.56	377.722	2.54E-02
33	CNR2	8.22	298.269	2.33E-02
34	SCARNA17	8.15	284.598	2.82E-02
35	ZNF583	8.14	281.602	3.82E-03
36	LOC100132111	7.93	244.331	4.17E-03
37	RORC	7.77	217.716	6.86E-06
38	ALOX5AP	7.60	193.662	9.59E-03
39	ROBO3	7.41	170.264	1.17E-02
40	CCL20	7.21	147.812	8.21E-08
41	UBE2D4	7.15	141.787	4.80E-02
42	MED12L	7.07	134.643	4.00E-02
43	LUCAT1	6.98	125.890	2.84E-02
44	C22orf23	6.93	122.178	4.58E-02
45	B3GALT2	6.92	121.387	2.09E-03

46	CD40LG	6.52	92.076	3.92E-04
47	COLQ	6.46	88.203	6.68E-04
48	PSMB8-AS1	6.45	87.458	4.15E-03
49	CCDC134	6.13	70.250	1.92E-02
50	CCL4	5.95	61.813	2.87E-04
51	GRID2IP	5.94	61.559	4.32E-02
52	EGR2	5.79	55.187	7.70E-04
53	KIT	5.73	53.033	1.01E-02
54	ADGRG5	5.73	52.900	7.12E-03
55	SLC4A10	5.71	52.260	6.54E-05
56	PDGFA	5.65	50.350	6.72E-03
57	THEMIS2	5.58	47.851	2.45E-02
58	DMKN	5.56	47.248	4.38E-02
59	KLRB1	5.38	41.696	6.34E-04
60	IL4I1	5.33	40.085	1.77E-03
61	NCR3	5.28	38.942	1.76E-05
62	NPIPB5	5.28	38.848	2.15E-02
63	LTA	5.15	35.422	5.89E-05
64	SOCS1	5.12	34.712	4.25E-02
65	LONRF1	5.03	32.662	2.56E-02
66	TIAM1	5.02	32.478	3.72E-03
67	TNF	4.97	31.342	1.03E-06
68	SLAMF1	4.92	30.280	4.92E-04
69	DUSP2	4.89	29.577	6.12E-04
70	PTGER2	4.74	26.752	1.97E-02
71	TNFSF14	4.73	26.494	2.27E-04
72	KLRG1	4.71	26.135	5.11E-05
73	UBASH3B	4.68	25.651	2.55E-02
74	LST1	4.61	24.463	4.40E-03
75	ARNTL	4.51	22.783	1.63E-02
76	COL5A3	4.41	21.262	5.82E-03
77	IL18RAP	4.40	21.067	5.17E-03
78	MFSD5	4.35	20.389	2.48E-02
79	USP30	4.28	19.377	4.73E-03
80	TNFSF8	4.20	18.347	5.29E-03
81	STX11	4.12	17.438	1.11E-03
82	JAKMIP1	4.09	17.069	1.52E-03
83	C17orf96	4.02	16.186	7.35E-03
84	SLFN12L	3.99	15.908	4.69E-02
85	HIVEP3	3.87	14.670	1.56E-02
86	CD69	3.86	14.527	1.69E-03
87	DUSP5	3.86	14.472	5.70E-03
88	ADAM12	3.80	13.951	1.62E-02
89	CACNB1	3.77	13.650	1.87E-02
90	TP53I11	3.76	13.547	4.16E-02
91	SYTL2	3.75	13.485	3.22E-03
92	PYROXD1	3.71	13.118	4.26E-03

93	MAF	3.70	12.977	6.10E-05
94	ZBTB16	3.69	12.884	2.29E-02
95	CCL3	3.61	12.205	4.64E-02
96	GRASP	3.59	12.043	2.75E-02
97	GNLY	3.57	11.852	3.65E-02
98	PTPN6	3.48	11.177	2.22E-05
99	ITGA1	3.46	11.032	5.46E-05
100	IL18R1	3.46	10.990	1.82E-03
101	GNG2	3.44	10.817	2.97E-04
102	ITGB7	3.37	10.336	8.08E-04
103	IL21R	3.36	10.290	4.68E-03
104	ABRACL	3.31	9.884	4.34E-02
105	FLT4	3.26	9.584	8.91E-03
106	TNFRSF9	3.26	9.559	1.71E-03
107	NR4A1	3.26	9.549	1.72E-03
108	EVI2A	3.26	9.549	4.72E-02
109	NINJ1	3.24	9.437	2.49E-03
110	SH2D1A	3.23	9.381	2.29E-02
111	CTSW	3.22	9.343	1.15E-04
112	JOSD2	3.21	9.257	2.28E-02
113	DPP4	3.18	9.058	2.72E-04
114	KCNK6	3.16	8.959	3.79E-02
115	JAML	3.13	8.740	2.70E-03
116	LTB	3.11	8.647	6.90E-04
117	TESK1	3.11	8.628	1.25E-02
118	EOMES	3.09	8.526	4.20E-02
119	BTG2	3.09	8.511	2.04E-03
120	RORA	3.08	8.455	3.17E-05
121	RUNX2	3.06	8.348	3.69E-02
122	PBX4	3.05	8.279	1.65E-02
123	IL15RA	3.05	8.276	4.39E-02
124	BTBD11	3.04	8.215	7.74E-03
125	AGAP2	3.03	8.163	4.14E-04
126	PIK3AP1	3.03	8.157	3.42E-03
127	ARHGAP31	3.02	8.095	4.50E-02
128	MDFIC	2.94	7.662	2.94E-04
129	APOBEC3G	2.89	7.404	3.28E-04
130	RASSF2	2.84	7.156	3.96E-04
131	C10orf54	2.80	6.968	8.54E-04
132	FOSL2	2.79	6.912	2.60E-03
133	DOK2	2.79	6.911	1.43E-03
134	IKZF2	2.78	6.856	3.90E-03
135	PRDM1	2.77	6.822	1.26E-03
136	TGFBR3	2.77	6.811	1.47E-04
137	RARG	2.77	6.808	4.15E-04
138	PLEK	2.76	6.769	8.57E-03
139	CD96	2.75	6.724	4.52E-04

140	IL12RB1	2.74	6.679	3.55E-04
141	FAS	2.72	6.572	1.84E-02
142	IL32	2.70	6.494	1.63E-05
143	LOC115110	2.70	6.488	4.07E-02
144	ZFP36L1	2.69	6.470	5.18E-06
145	TBX21	2.66	6.303	7.31E-03
146	SCRN1	2.65	6.285	8.16E-03
147	FEZ1	2.65	6.284	7.09E-04
148	MYO1G	2.64	6.243	1.20E-03
149	SCART1	2.63	6.202	2.47E-03
150	RGS3	2.63	6.182	2.84E-03
151	LPAR2	2.60	6.061	1.50E-02
152	PTGER4	2.58	5.984	9.23E-03
153	HIC1	2.57	5.956	8.57E-04
154	SH2D2A	2.57	5.946	2.40E-05
155	MAP3K8	2.56	5.910	4.99E-02
156	LPXN	2.54	5.832	1.47E-03
157	IFNGR1	2.54	5.809	9.93E-05
158	CDS2	2.54	5.804	2.62E-02
159	SLC37A1	2.54	5.803	2.15E-02
160	OFD1	2.53	5.793	4.02E-02
161	TRAF4	2.52	5.718	1.83E-02
162	KDM6B	2.51	5.707	2.39E-04
163	SLA	2.51	5.680	4.35E-03
164	TAGAP	2.50	5.665	8.03E-03
165	RELB	2.48	5.586	9.65E-04
166	CD274	2.48	5.569	3.65E-02
167	CEBPD	2.45	5.459	1.37E-02
168	TARP	2.45	5.448	2.64E-04
169	PDE4D	2.41	5.328	1.23E-03
170	CD82	2.40	5.285	8.89E-04
171	NFKB1	2.40	5.274	5.68E-05
172	PLXNC1	2.33	5.014	6.10E-03
173	ACAP3	2.29	4.888	2.91E-02
174	SP4	2.29	4.884	1.55E-02
175	CCR5	2.26	4.793	1.39E-03
176	ITGB2	2.26	4.784	2.84E-04
177	PRDM8	2.25	4.764	3.09E-02
178	GIT1	2.24	4.738	4.00E-03
179	PHLDA1	2.24	4.727	2.96E-03
180	NKG7	2.24	4.708	1.79E-02
181	IL2RB	2.23	4.692	1.95E-04
182	ZMIZ2	2.23	4.677	1.42E-03
183	CORO1A	2.22	4.662	7.21E-05
184	PAG1	2.22	4.654	5.97E-05
185	IRAK2	2.21	4.629	4.15E-02
186	LAG3	2.21	4.629	2.47E-02

187	KIF21B	2.21	4.626	1.93E-03
188	FLI1	2.20	4.594	2.69E-02
189	ARHGAP30	2.20	4.591	8.08E-05
190	REL	2.20	4.591	1.30E-04
191	ZC3H12A	2.19	4.575	1.00E-03
192	CASP1	2.17	4.511	2.09E-02
193	ZNF600	2.17	4.501	3.15E-02
194	FOXO1	2.16	4.483	6.33E-03
195	TP53	2.16	4.466	2.06E-02
196	TTC39C	2.15	4.432	9.09E-04
197	ELMO2	2.14	4.411	6.07E-03
198	PPM1M	2.14	4.406	1.46E-03
199	CELF2	2.14	4.403	1.63E-04
200	MPZL3	2.13	4.370	6.24E-03
201	RARA	2.12	4.361	2.98E-03
202	BCL6	2.12	4.358	8.36E-03
203	SATB1	2.10	4.291	3.10E-04
204	CAMK2D	2.09	4.263	5.96E-03
205	IFFO2	2.09	4.250	1.50E-02
206	BIRC3	2.08	4.220	1.02E-04
207	PRF1	2.06	4.184	1.75E-04
208	PHF11	2.06	4.167	4.74E-02
209	RECQL	2.06	4.165	5.54E-03
210	CD6	2.06	4.160	4.26E-04
211	SLC41A1	2.05	4.143	3.11E-02
212	FKBP11	2.05	4.141	3.02E-03
213	SLC16A3	2.04	4.126	9.31E-03
214	TNFAIP3	2.04	4.119	7.87E-04
215	GPR65	2.04	4.104	1.16E-03
216	PTPN22	2.04	4.103	1.96E-03
217	FMNL3	2.02	4.057	1.74E-03
218	FAM129A	2.01	4.038	5.79E-03
219	KIF5C	2.01	4.038	4.14E-03
220	DENND3	2.00	4.003	2.20E-02
221	NCBP3	2.00	3.992	2.74E-02
222	PHC1	2.00	3.989	5.24E-03
223	RBPJ	1.99	3.972	1.27E-04
224	HIF1A	1.99	3.967	1.88E-04
225	VAMP1	1.98	3.951	9.60E-03
226	LOC284454	1.96	3.891	4.02E-02
227	CARMIL2	1.96	3.887	4.29E-04
228	C21orf91	1.95	3.862	6.96E-03
229	TNFRSF25	1.95	3.861	4.58E-04
230	NFKBIA	1.95	3.852	8.46E-05
231	BTG1	1.93	3.821	3.94E-04
232	FLT3LG	1.93	3.812	2.49E-02
233	TRAF1	1.92	3.784	4.11E-04

234	IRF9	1.92	3.782	1.79E-02
235	LAX1	1.92	3.779	2.37E-02
236	SYT11	1.90	3.741	3.10E-02
237	FAM168A	1.89	3.711	2.13E-02
238	BCL2	1.88	3.690	4.38E-03
239	LCP2	1.88	3.685	2.88E-03
240	TNFRSF14	1.87	3.652	3.28E-02
241	SLC25A22	1.85	3.615	4.94E-02
242	PRNP	1.84	3.574	1.94E-03
243	CYLD	1.83	3.567	1.62E-02
244	GMIP	1.83	3.564	2.57E-03
245	SLAMF7	1.83	3.556	4.03E-02
246	FYCO1	1.83	3.550	1.74E-02
247	POU2F2	1.82	3.540	1.60E-02
248	CASP4	1.82	3.530	5.21E-03
249	VPS13B	1.82	3.525	3.70E-02
250	ZNF611	1.81	3.518	3.40E-02
251	SSH1	1.81	3.513	2.78E-03
252	PLK3	1.81	3.513	3.10E-03
253	42979	1.81	3.510	8.39E-03
254	DENND2D	1.81	3.495	1.22E-02
255	DHRS7	1.80	3.473	3.09E-02
256	METTL16	1.79	3.468	2.36E-02
257	CD44	1.79	3.448	1.23E-03
258	SSH2	1.78	3.423	1.80E-02
259	PREX1	1.77	3.422	2.51E-03
260	STAT4	1.77	3.412	1.75E-03
261	UFM1	1.77	3.401	9.43E-03
262	RBMS1	1.76	3.396	1.19E-02
263	SH3BP2	1.76	3.388	2.87E-03
264	MAFK	1.76	3.387	2.95E-02
265	DUSP10	1.76	3.384	4.17E-02
266	IGFLR1	1.76	3.383	2.88E-02
267	DNMT3A	1.75	3.366	1.62E-03
268	FMNL1	1.75	3.354	5.00E-04
269	PTPRJ	1.74	3.340	3.80E-02
270	CAPN15	1.74	3.338	4.00E-03
271	RNF19A	1.74	3.335	4.35E-04
272	SPOCK2	1.73	3.319	2.76E-03
273	MOB3A	1.73	3.319	3.87E-03
274	CD2	1.72	3.304	2.08E-03
275	MLLT6	1.72	3.303	2.21E-04
276	CBLB	1.72	3.302	3.20E-02
277	EMB	1.72	3.302	3.64E-03
278	ERVK13-1	1.72	3.297	2.88E-02
279	IFI6	1.70	3.258	4.19E-02
280	NFKB2	1.70	3.258	4.93E-03

281	WWC3	1.70	3.256	2.35E-02
282	TJAP1	1.70	3.244	3.28E-02
283	GADD45B	1.70	3.241	1.35E-02
284	RNH1	1.69	3.237	1.16E-02
285	GID8	1.68	3.208	3.29E-02
286	HELZ2	1.68	3.208	4.74E-02
287	FBXO41	1.68	3.206	4.72E-03
288	SH3KBP1	1.68	3.201	8.80E-03
289	PHACTR2	1.67	3.181	5.31E-03
290	GPR171	1.67	3.176	2.73E-03
291	VAV1	1.67	3.173	1.05E-02
292	BACH2	1.67	3.172	1.05E-02
293	INO80D	1.66	3.170	3.11E-03
294	ICAM3	1.66	3.159	6.55E-03
295	FOXJ2	1.66	3.154	3.28E-02
296	JAK3	1.66	3.151	2.02E-03
297	ABCC10	1.64	3.125	2.50E-02
298	LCP1	1.64	3.124	2.75E-03
299	RALGAPA2	1.64	3.113	3.57E-02
300	CD52	1.64	3.112	5.21E-03
301	DUSP4	1.64	3.106	3.96E-03
302	RUNX3	1.63	3.104	1.22E-02
303	ANKRD44	1.63	3.099	5.83E-03
304	RRM2B	1.63	3.097	3.27E-02
305	LFNG	1.63	3.085	6.75E-03
306	PIK3C2A	1.62	3.066	1.79E-02
307	ADGRE5	1.61	3.056	1.80E-03
308	ACADVL	1.61	3.048	8.30E-04
309	ARL4C	1.60	3.027	5.73E-03
310	C19orf66	1.59	3.020	2.72E-02
311	ARHGAP26	1.59	3.003	1.40E-02
312	IL2RG	1.58	2.992	6.96E-03
313	MAP2K4	1.58	2.983	2.55E-02
314	MYO1F	1.58	2.980	3.26E-02
315	NCK2	1.57	2.977	4.09E-02
316	TNFRSF1B	1.56	2.951	7.40E-04
317	TRAK2	1.55	2.937	1.61E-02
318	GBP5	1.55	2.931	6.12E-03
319	PIK3R5	1.55	2.929	3.87E-02
320	ELMSAN1	1.55	2.924	1.95E-02
321	PPP1R16B	1.55	2.922	1.82E-02
322	TSPYL2	1.55	2.918	6.37E-03
323	WIPF2	1.54	2.915	3.59E-02
324	RASSF5	1.54	2.905	2.29E-02
325	FNBP1	1.54	2.904	2.91E-03
326	RFLNB	1.54	2.903	3.39E-02
327	EFHD2	1.53	2.891	5.18E-03

328	ETS1	1.53	2.890	1.12E-03
329	ZNFX1	1.53	2.882	3.05E-02
330	RASGRP1	1.52	2.876	3.23E-02
331	PSME2	1.52	2.871	1.66E-03
332	LTBP3	1.52	2.871	2.46E-02
333	RPS6KA3	1.52	2.871	6.77E-04
334	STAT6	1.52	2.859	2.27E-03
335	PLEKHG2	1.51	2.848	1.83E-02
336	TESPA1	1.51	2.841	1.81E-02
337	FURIN	1.50	2.826	2.14E-03
338	PGGHG	1.50	2.826	2.94E-04
339	GLS	1.50	2.822	5.33E-03
340	CERK	1.49	2.807	5.62E-03
341	MEX3C	1.48	2.786	2.49E-02
342	CYTIP	1.48	2.782	3.04E-02
343	RAB8B	1.47	2.780	2.75E-03
344	ABR	1.47	2.776	4.62E-02
345	ARFGAP3	1.47	2.773	1.79E-02
346	VPS16	1.47	2.772	4.74E-02
347	MICAL2	1.47	2.771	3.82E-02
348	ASH1L	1.47	2.769	1.02E-02
349	NPTN	1.46	2.757	3.31E-02
350	EDEM1	1.46	2.754	3.34E-02
351	CCDC88B	1.46	2.745	2.59E-03
352	LYST	1.46	2.743	8.79E-03
353	NCS1	1.45	2.741	1.84E-02
354	NFAT5	1.45	2.729	9.28E-03
355	KAT2B	1.45	2.725	8.68E-03
356	SMG6	1.44	2.712	1.29E-02
357	STIM2	1.43	2.697	2.65E-02
358	MGRN1	1.43	2.690	4.62E-02
359	CLASP1	1.42	2.683	2.27E-02
360	INPP5D	1.42	2.670	4.33E-02
361	AHR	1.42	2.669	4.33E-02
362	ABCA2	1.40	2.647	1.72E-02
363	ABHD17A	1.40	2.647	6.05E-03
364	KLF13	1.39	2.630	2.69E-03
365	PARP8	1.39	2.627	1.33E-02
366	OSBPL3	1.39	2.622	5.58E-03
367	IER5	1.38	2.606	4.08E-02
368	NFKBIZ	1.38	2.605	8.46E-03
369	ZHX2	1.37	2.576	3.91E-02
370	APMAP	1.36	2.575	3.47E-02
371	MYH9	1.36	2.569	2.81E-04
372	CARD11	1.36	2.565	9.47E-03
373	KCNAB2	1.36	2.564	1.20E-02
374	BHLHE40	1.36	2.558	1.33E-02

375	TUBGCP6	1.35	2.556	1.13E-02
376	SRGN	1.35	2.550	1.48E-03
377	WHRN	1.34	2.530	2.25E-02
378	IL27RA	1.33	2.515	1.58E-02
379	DNPEP	1.33	2.510	2.35E-02
380	CBX6	1.32	2.501	6.40E-03
381	MATK	1.32	2.501	3.22E-02
382	PFKFB3	1.32	2.498	2.89E-02
383	DOCK2	1.32	2.497	8.37E-03
384	SMAD3	1.32	2.492	2.20E-02
385	FAM126B	1.32	2.490	4.33E-02
386	SEC24D	1.32	2.488	2.85E-02
387	STK17B	1.31	2.488	3.12E-03
388	CD247	1.30	2.466	3.01E-02
389	PELO	1.30	2.457	4.73E-02
390	CTSA	1.29	2.450	3.84E-02
391	GALNT10	1.29	2.447	4.83E-02
392	C9orf69	1.29	2.442	4.98E-02
393	ZSWIM8	1.29	2.438	1.65E-02
394	ZC3H4	1.29	2.437	3.92E-02
395	VPS13C	1.28	2.425	3.47E-03
396	CREBBP	1.27	2.414	1.16E-02
397	HERPUD1	1.27	2.409	3.48E-02
398	MEF2D	1.26	2.402	1.69E-02
399	AGL	1.26	2.401	3.70E-02
400	ITGAL	1.26	2.400	1.50E-02
401	ARL6IP5	1.26	2.400	2.35E-02
402	AKNA	1.25	2.385	3.01E-02
403	PMM2	1.25	2.384	2.98E-02
404	CD8A	1.25	2.374	1.44E-02
405	DUSP16	1.25	2.373	4.56E-02
406	MYBBP1A	1.25	2.370	2.29E-02
407	SOS2	1.24	2.369	4.33E-02
408	TACC1	1.24	2.367	8.97E-03
409	HLA-B	1.24	2.360	5.37E-03
410	PTAR1	1.24	2.358	4.83E-02
411	ID2	1.24	2.356	3.47E-02
412	CSK	1.24	2.355	8.16E-03
413	FLNA	1.23	2.350	4.99E-03
414	TMEM185B	1.23	2.344	3.95E-02
415	CCL5	1.23	2.343	3.29E-02
416	ITK	1.22	2.335	3.80E-02
417	CDK17	1.22	2.333	3.28E-02
418	CPD	1.22	2.332	4.11E-02
419	SLFN11	1.21	2.313	3.58E-02
420	CD53	1.21	2.312	1.01E-02
421	PLCB2	1.21	2.310	4.75E-02

422	PIK3CD	1.21	2.306	9.53E-03
423	MGAT1	1.20	2.305	1.75E-02
424	EP300	1.20	2.305	4.93E-02
425	HMGN4	1.20	2.299	1.98E-02
426	EFCAB14	1.20	2.293	1.27E-02
427	EML4	1.19	2.278	2.84E-02
428	PPP1R9B	1.19	2.274	1.06E-02
429	SLC38A10	1.18	2.258	2.79E-02
430	BST2	1.17	2.255	2.93E-02
431	HLA-C	1.17	2.255	4.77E-03
432	VPS4B	1.17	2.250	3.96E-02
433	RNF213	1.16	2.240	1.84E-03
434	TC2N	1.16	2.236	1.47E-02
435	KPNA3	1.15	2.225	4.04E-02
436	EFR3A	1.15	2.214	2.85E-02
437	WDR55	1.15	2.213	2.16E-02
438	ARPC2	1.15	2.213	3.07E-03
439	PREB	1.15	2.213	4.82E-02
440	BTAF1	1.15	2.212	1.68E-02
441	USP19	1.14	2.206	2.52E-02
442	ANXA6	1.14	2.198	7.19E-03
443	PLEC	1.13	2.193	4.04E-03
444	GBP2	1.13	2.183	2.21E-02
445	MYSM1	1.12	2.179	3.76E-02
446	PPP1R10	1.12	2.168	4.60E-02
447	PSME1	1.12	2.167	1.30E-02
448	LBH	1.11	2.158	2.55E-02
449	CHPF2	1.11	2.154	4.45E-02
450	PPP4R3A	1.11	2.153	3.25E-02
451	SEMA4D	1.10	2.142	1.65E-02
452	NR3C1	1.09	2.121	2.09E-02
453	HIF1AN	1.08	2.119	4.52E-02
454	CAD	1.08	2.115	1.32E-02
455	GOLGA8B	1.08	2.114	2.94E-02
456	LCK	1.08	2.112	1.43E-02
457	EXOC7	1.08	2.111	4.07E-02
458	B4GALT1	1.08	2.109	1.31E-02
459	HLA-A	1.08	2.107	2.27E-02
460	SERPINB9	1.07	2.106	3.29E-02
461	CRTC2	1.07	2.105	3.17E-02
462	ARHGEF6	1.07	2.101	4.04E-02
463	MDM2	1.07	2.098	2.13E-02
464	ATN1	1.06	2.090	3.66E-02
465	LAPTM5	1.06	2.089	1.92E-02
466	KIF21A	1.06	2.088	3.64E-02
467	MAP7D1	1.05	2.077	2.16E-02
468	TSC2	1.05	2.074	4.59E-02

469	PRKAR1A	1.05	2.072	9.25E-03
470	BCL9L	1.05	2.070	6.09E-03
471	PPRC1	1.05	2.069	2.92E-02
472	SEL1L	1.05	2.064	4.54E-02
473	RAC2	1.05	2.064	1.19E-02
474	PIP4K2B	1.04	2.061	3.73E-02
475	PITPNM1	1.04	2.057	2.07E-02
476	FMR1	1.04	2.053	4.49E-02
477	TMEM123	1.04	2.053	5.05E-03
478	DGKA	1.04	2.052	2.37E-02
479	SLC7A1	1.03	2.049	3.20E-02
480	PRKCA	1.03	2.036	4.72E-02
481	PKM	1.03	2.035	4.50E-03
482	SAMD9	1.02	2.035	3.14E-02
483	SFMBT2	1.02	2.031	4.90E-02
484	NEK7	1.02	2.030	4.99E-02
485	SLC7A5	1.02	2.024	2.26E-02
486	DAZAP2	1.01	2.013	7.24E-03
487	TNFAIP8	1.01	2.008	1.99E-02
488	IGF2R	1.00	2.003	1.90E-02
489	WIPF1	1.00	2.001	4.39E-02
490	USP4	1.00	1.995	2.09E-02
491	TAP2	1.00	1.993	7.47E-03
492	DDX55	0.99	1.993	4.57E-02
493	RHOG	0.99	1.989	3.41E-02
494	JAK1	0.99	1.986	1.24E-02
495	PARP14	0.99	1.982	1.98E-02
496	IKZF1	0.98	1.979	1.06E-02
497	BAP1	0.98	1.976	4.59E-02
498	KIAA1551	0.98	1.975	1.18E-02
499	CLDND1	0.98	1.974	2.97E-02
500	SPTAN1	0.98	1.974	1.06E-02
501	IL4R	0.98	1.974	2.09E-02
502	FUBP1	0.98	1.967	9.04E-03
503	TYK2	0.97	1.963	9.23E-03
504	YTHDF3	0.97	1.961	4.27E-02
505	KANSL1	0.97	1.960	4.44E-02
506	DNAJC3	0.96	1.951	3.99E-02
507	NUP62	0.96	1.946	3.22E-02
508	KMT2B	0.96	1.942	4.05E-02
509	KMT2D	0.96	1.940	1.15E-02
510	CCND2	0.95	1.936	4.21E-02
511	KIF3B	0.95	1.930	3.75E-02
512	CD74	0.95	1.928	1.65E-02
513	RAPGEF6	0.95	1.926	4.32E-02
514	MED24	0.95	1.925	3.12E-02
515	UTP20	0.94	1.920	4.21E-02

516	ICAM1	0.94	1.920	4.83E-02
517	TXNRD1	0.94	1.917	1.12E-02
518	STK4	0.93	1.910	4.70E-02
519	XIST	0.93	1.901	2.75E-02
520	PURB	0.92	1.898	3.01E-02
521	NEAT1	0.92	1.896	2.01E-02
522	ACSL4	0.92	1.893	4.58E-02
523	TAX1BP1	0.92	1.892	1.71E-02
524	LOC100190986	0.91	1.885	4.21E-02
525	TLN1	0.91	1.879	1.92E-02
526	NOTCH1	0.91	1.877	3.46E-02
527	ANXA5	0.91	1.876	3.16E-02
528	SH3BGRL3	0.90	1.872	4.41E-02
529	SELENOT	0.90	1.871	4.57E-02
530	MYO9B	0.90	1.862	3.77E-02
531	SQSTM1	0.90	1.861	1.53E-02
532	ITPR3	0.89	1.859	1.20E-02
533	TNRC18	0.89	1.848	2.95E-02
534	SEC23B	0.88	1.841	3.09E-02
535	KLF6	0.88	1.838	2.38E-02
536	AHNAK	0.88	1.838	2.15E-02
537	42985	0.88	1.837	1.79E-02
538	SLC5A3	0.87	1.833	4.91E-02
539	RAPGEF1	0.87	1.829	4.25E-02
540	SRCAP	0.86	1.818	1.28E-02
541	SPEN	0.86	1.813	2.45E-02
542	FLII	0.86	1.812	3.78E-02
543	STAT3	0.85	1.804	2.91E-02
544	ARHGDIB	0.85	1.798	4.22E-02
545	TPT1	0.84	1.793	5.98E-03
546	SRPRA	0.84	1.791	3.23E-02
547	ANKRD12	0.84	1.790	2.81E-02
548	MBD1	0.84	1.790	3.63E-02
549	IDS	0.84	1.787	4.97E-02
550	EHD1	0.84	1.787	4.80E-02
551	NUP98	0.83	1.784	3.92E-02
552	PSD4	0.83	1.775	4.93E-02
553	NCBP2	0.81	1.753	3.78E-02
554	B2M	0.81	1.749	3.46E-02
555	EVL	0.79	1.729	4.65E-02
556	CLINT1	0.79	1.727	4.91E-02
557	TGOLN2	0.78	1.722	2.48E-02
558	MACF1	0.78	1.711	2.55E-02
559	TMEM30A	0.77	1.711	3.14E-02
560	KDM2A	0.77	1.702	3.41E-02
561	ZC3HAV1	0.75	1.681	2.13E-02
562	PTPRC	0.74	1.670	4.23E-02

563	TAPBP	0.74	1.665	3.33E-02
564	SLC3A2	0.73	1.661	4.83E-02
565	ATP8B2	0.73	1.654	4.13E-02
566	ARID1A	0.73	1.653	3.51E-02
567	SEC61A1	0.70	1.628	3.80E-02
568	GOLGA3	0.70	1.619	4.53E-02
569	VPS13A	0.69	1.615	4.60E-02
570	ACTB	0.62	1.537	2.81E-02
571	MRFAP1	-0.63	0.647	4.75E-02
572	CCT3	-0.68	0.623	3.91E-02
573	MR1	-0.69	0.619	2.31E-02
574	USP48	-0.72	0.609	4.22E-02
575	EI24	-0.72	0.607	4.51E-02
576	CLNS1A	-0.75	0.594	3.57E-02
577	SDHA	-0.79	0.578	3.54E-02
578	RPS10	-0.80	0.576	4.57E-02
579	HMGA1	-0.82	0.568	2.78E-02
580	MSH6	-0.82	0.567	3.56E-02
581	DLD	-0.84	0.560	2.96E-02
582	MRPS27	-0.84	0.557	4.91E-02
583	YIF1B	-0.86	0.551	3.54E-02
584	ATP5I	-0.87	0.547	4.69E-02
585	UQCRC2	-0.87	0.546	4.20E-02
586	MCM6	-0.88	0.544	2.60E-02
587	UBE2S	-0.90	0.536	4.09E-02
588	FEN1	-0.90	0.536	4.02E-02
589	MCFD2	-0.91	0.534	2.63E-02
590	VAPA	-0.92	0.529	2.55E-02
591	ASH2L	-0.92	0.528	2.85E-02
592	USMG5	-0.93	0.524	4.14E-02
593	HMGCS1	-0.94	0.521	2.04E-02
594	CLPP	-0.94	0.521	4.06E-02
595	PSMB1	-0.94	0.520	2.42E-02
596	ATP6V1F	-0.99	0.505	4.77E-02
597	NDUFA1	-1.06	0.481	3.04E-02
598	MARCKSL1	-1.06	0.480	3.70E-02
599	GALNT2	-1.06	0.480	3.85E-02
600	FLOT1	-1.06	0.479	2.25E-02
601	PCCB	-1.09	0.470	4.87E-02
602	ACER3	-1.10	0.467	4.46E-02
603	RHOBTB3	-1.11	0.464	1.91E-02
604	UBAC1	-1.12	0.461	1.29E-02
605	RUVBL1	-1.12	0.459	4.07E-02
606	SREBF1	-1.13	0.456	1.45E-02
607	MAD2L1	-1.13	0.456	4.58E-02
608	NARF	-1.14	0.454	1.56E-02
609	PARN	-1.16	0.449	2.77E-02

610	HN1L	-1.16	0.448	2.54E-02
611	IDH1	-1.17	0.444	1.69E-02
612	RPRD1A	-1.19	0.439	4.88E-02
613	GADD45GIP1	-1.19	0.438	4.31E-02
614	FUCA2	-1.20	0.436	2.23E-02
615	COTL1	-1.20	0.435	2.47E-02
616	EPHB4	-1.21	0.432	4.32E-02
617	CLSPN	-1.21	0.432	2.64E-02
618	MKI67	-1.21	0.432	4.28E-02
619	ORC1	-1.21	0.431	3.00E-02
620	ARHGAP21	-1.22	0.430	2.85E-02
621	ASAH1	-1.24	0.425	4.36E-02
622	ACTL6A	-1.24	0.424	3.81E-02
623	PDLIM5	-1.26	0.417	2.59E-02
624	CHP1	-1.29	0.410	3.84E-02
625	JUP	-1.30	0.407	2.63E-02
626	SSBP3	-1.31	0.403	4.66E-03
627	ABCB10	-1.32	0.401	1.38E-02
628	HBS1L	-1.32	0.399	1.99E-02
629	DHCR24	-1.33	0.398	3.39E-02
630	ACSL6	-1.35	0.392	2.78E-02
631	MINOS1	-1.38	0.385	2.44E-02
632	XPNPEP3	-1.39	0.381	2.71E-02
633	MCUR1	-1.39	0.380	2.86E-02
634	APOE	-1.41	0.376	3.41E-02
635	ADK	-1.42	0.374	2.07E-02
636	C19orf48	-1.42	0.373	2.88E-02
637	SLC29A1	-1.43	0.372	2.27E-02
638	TRAM2	-1.43	0.372	4.38E-02
639	ZNF714	-1.43	0.372	3.29E-02
640	FYB	-1.44	0.369	7.40E-03
641	ACSS2	-1.44	0.369	1.90E-02
642	RRM2	-1.46	0.363	2.59E-02
643	MTX2	-1.46	0.362	2.55E-02
644	TMEM97	-1.48	0.358	1.45E-02
645	PKP4	-1.49	0.356	4.17E-02
646	H19	-1.50	0.353	2.78E-02
647	HIST1H2AC	-1.52	0.349	2.21E-02
648	TMEM41A	-1.52	0.348	1.97E-02
649	LINC01578	-1.55	0.342	3.80E-02
650	CTCFL	-1.59	0.333	2.76E-02
651	ASPH	-1.60	0.331	4.17E-02
652	QPCTL	-1.61	0.328	2.49E-02
653	NDUFA7	-1.61	0.327	4.19E-02
654	HIST1H2BK	-1.62	0.326	2.92E-02
655	CDT1	-1.65	0.320	8.52E-03
656	PRR11	-1.65	0.320	4.48E-02

657	KRT8	-1.67	0.314	1.43E-02
658	ECT2	-1.68	0.312	1.28E-02
659	TXNRD2	-1.69	0.310	4.61E-02
660	GTF2H3	-1.71	0.306	2.40E-02
661	CHML	-1.71	0.306	3.10E-02
662	PRC1	-1.72	0.303	4.33E-02
663	SORD	-1.74	0.300	3.13E-02
664	PLK1	-1.76	0.296	1.68E-02
665	GMNN	-1.76	0.295	4.18E-02
666	FXN	-1.79	0.289	1.68E-02
667	SKA2	-1.81	0.284	2.60E-02
668	SNORD10	-1.83	0.281	4.02E-02
669	RASA3	-1.92	0.264	2.67E-03
670	HAPLN3	-1.92	0.264	1.64E-02
671	SNORA73B	-1.92	0.264	7.96E-03
672	ITGB5	-1.93	0.262	4.54E-02
673	SHROOM1	-2.00	0.250	4.70E-02
674	STIL	-2.00	0.250	3.26E-02
675	ZNF445	-2.03	0.245	2.39E-03
676	TGM2	-2.03	0.244	1.70E-02
677	TYMS	-2.05	0.241	4.15E-03
678	MRPL40	-2.06	0.239	2.54E-02
679	SLC25A13	-2.12	0.231	3.50E-02
680	TATDN3	-2.13	0.228	1.79E-02
681	TMEM107	-2.19	0.219	1.76E-02
682	SLC25A4	-2.22	0.215	3.96E-02
683	GLB1L2	-2.31	0.202	3.55E-02
684	LOC101928118	-2.31	0.202	3.59E-02
685	AFMID	-2.35	0.197	1.91E-02
686	KDELC2	-2.48	0.180	4.53E-03
687	ALPK1	-2.63	0.162	3.14E-02
688	TRABD2A	-2.90	0.134	2.23E-02
689	ZNF471	-2.99	0.126	2.44E-02
690	SESN3	-3.00	0.125	8.92E-03
691	CCR7	-3.19	0.109	2.75E-03
692	HBA2	-3.29	0.102	3.62E-02
693	CDC6	-3.40	0.095	3.01E-02
694	LINC00923	-4.73	0.038	1.19E-02
695	ABCA9	-5.29	0.026	1.69E-02
696	SPATA7	-5.42	0.023	4.28E-02
697	LIMCH1	-5.66	0.020	3.94E-02
698	TMEM254-AS1	-5.79	0.018	1.45E-02
699	SMCO4	-6.12	0.014	4.40E-02
700	POM121L8P	-6.60	0.010	4.73E-02
701	ZNF70	-6.64	0.010	4.07E-02
702	DHRS2	-6.86	0.009	4.15E-02
703	ZIM2-AS1	-6.99	0.008	4.64E-02

704	PAWR	-7.54	0.005	3.54E-02
705	ZSCAN21	-7.92	0.004	4.93E-02
706	SLC30A4	-8.01	0.004	4.53E-02
707	DISP2	-8.25	0.003	4.87E-02
708	TPTE2P3	-8.29	0.003	3.28E-02
709	LOC102724927	-8.40	0.003	4.59E-02
710	TMEM220	-8.74	0.002	3.61E-02
711	GRB14	-8.78	0.002	4.66E-02
712	ALPP	-8.98	0.002	4.69E-02
713	ELMOD1	-9.06	0.002	2.77E-02
714	CACNA1C-AS2	-9.07	0.002	4.94E-02
715	KIAA1210	-9.13	0.002	2.01E-02
716	TMEM132E	-9.17	0.002	4.98E-02
717	C19orf35	-9.19	0.002	1.41E-02
718	KIF1A	-9.27	0.002	4.69E-02
719	TLR10	-9.43	0.001	3.00E-02
720	TRIQK	-9.46	0.001	2.20E-02
721	WIPF3	-9.54	0.001	3.56E-02
722	LGR6	-9.56	0.001	3.80E-02
723	LOC646736	-9.57	0.001	3.03E-02
724	DUSP28	-9.60	0.001	4.34E-02
725	COL28A1	-9.65	0.001	4.46E-02
726	LOC283045	-9.67	0.001	4.50E-02
727	ROR1	-9.71	0.001	3.71E-02
728	LAMA2	-9.79	0.001	4.14E-02
729	CFAP43	-9.88	0.001	3.39E-02
730	MAGEB10	-9.91	0.001	3.46E-02
731	SUSD5	-9.93	0.001	3.05E-02
732	IL10	-9.97	0.001	3.48E-02
733	NRCAM	-9.97	0.001	1.14E-02
734	DEPDC7	-9.98	0.001	3.09E-02
735	42981	-9.98	0.001	4.47E-02
736	PRRT3	-10.04	0.001	2.76E-02
737	C2orf27A	-10.11	0.001	4.60E-02
738	GBAP1	-10.12	0.001	4.49E-02
739	KLHL7-AS1	-10.13	0.001	1.75E-02
740	ASIC1	-10.20	0.001	4.64E-02
741	HCG27	-10.21	0.001	4.39E-02
742	IRGM	-10.40	0.001	3.84E-02
743	FBXO36	-10.52	0.001	4.72E-02
744	RAB37	-10.53	0.001	1.77E-02
745	FSIP1	-10.56	0.001	2.54E-02
746	TSGA10	-10.81	0.001	3.03E-02
747	CSMD2	-10.92	0.001	2.81E-02
748	B3GAT1	-11.35	0.000	1.58E-02
749	ZNF425	-11.37	0.000	7.83E-03
